# Associations of heat exposure with mental health and suicide in children and adolescents: a systematic review and meta-analysis

**DOI:** 10.1038/s44184-026-00190-w

**Published:** 2026-01-30

**Authors:** Ka Yan Lai, Sarah Bauermeister, Chinmoy Sarkar

**Affiliations:** 1 Healthy High Density Cities Lab, HKUrbanLab, The University of Hong Kong, Hong Kong Special Administrative Region, China; 2 Department of Urban Planning & Design, Faculty of Architecture, The University of Hong Kong, Hong Kong Special Administrative Region, China; 3 Institute for Climate and Carbon Neutrality, The University of Hong Kong, Hong Kong Special Administrative Region, China; 4 Department of Psychiatry, University of Oxford, Oxford, United Kingdom

**Keywords:** Climate change, Psychiatric disorders, Environmental sciences, Environmental social sciences, Scientific community, Social sciences

## Abstract

Limited evidence exists on the association between high temperature and mental health in younger age group. We conducted a systematic search on PubMed, Web of Science, CINAHL Plus and PsycInfo and reviewed studies that explore the associations of heat exposure with mental health and suicide among children and adolescents ( ≤24 years). Twenty-eight observational studies published over 2007-2025 were included for systematic review. Exposure to high temperature was associated with 13% (95% confidence intervals: 1.08 to 1.19; *n* = 720,512) higher risk of hospital visits or hospitalizations for mental health disorders, 14% (1.01 to 1.28; *n* = 529,654) higher risk for schizophrenia, 18% (1.03 to 1.34; *n* = 146,046) higher risk for depression, and 12% (1.06 to 1.18; *n* = 1,188,501) higher risk for composite mental health illnesses, relative to low temperature. Each 1 °C increment in temperature was associated with 1.0% (1.00 to 1.02; *n* = 30,749) higher risk of suicide. Findings suggest the need for early life interventions and evidence-based adaptation policies for preventing against climate-induced mental illnesses and suicide. Optimizing policies will be important for high- and upper-middle-income countries, while more scientific evidence from lower-income economies are warranted.

## Introduction

Mental health disorders constitute a significant proportion of global burden of disease among children and adolescents, affecting childhood development and wellbeing, transition to adulthood, and overall development across the lifespan^1,2^. The estimated global mean prevalence of mental disorders among individuals aged between 5 and 24 years reached 11.6% in 2019, equivalent to 293 million people^3^. It has been estimated that 24.9% of the total number of years lived with disability attributed to mental disorders were recorded before an individual reached 25 years of age^3^. Early life mental disorders including depression have been found to be associated with higher risks of recurrence in adulthood, as well as increasing risk of interpersonal difficulties, cardiovascular disease, drug use, and suicidal behaviors in later life^4,5^. Given the stigma associated with mental health, identifying early life environmental and social determinants of mental health remains a global priority towards timely treatments and interventions for strengthening psychological resilience across the life span^6^.

Climatic extremes have emerged as a new norm with the 10 warmest years in the 143-year global climatic archive being recorded since 2010^7^ and constitute important risk factors of mental health disorders^8–10^. Globally, children are disproportionately affected by extreme heat^11^, with 466 million estimated to experience at least twice the number of extreme hot days over 2020–24 as compared to their predecessors six decades ago^12^. However, little is known about the climate-induced mental health outcomes in children and adolescents. It has been suggested that climate change-related rapid temperature anomalies such as higher frequency, intensity and duration of heat extremes may induce direct stress, impairing mental health of children and adolescents^13^. Indirectly, children and adolescents with greater awareness of climate change have been shown to have a higher likelihood of developing climate anxiety^14^ and solastalgia^15^. The younger age group is more vulnerable to the indirect impacts of high temperature exposures due to their higher biological sensitivity, limited adaptability and greater reliance on caregivers^16^. High temperature exposures may also reduce outdoor time and physical activity among younger age group with lower capacity for thermoregulation, resulting in sedentary lifestyle, reduced body fitness and elevated risk of mental illness^17^. Particularly, elevated temperatures heat-up outdoor playgrounds placed with dark-colored wet-pour rubber and synthetic turf. This in turn increases risks for burn injuries, which may discourage stress-relieving outdoor opportunities^18^. Hot weather may also serve as an environmental disruptor diminishing sleep quality, affect daily living and mental well-being^19^. Physiologically, heat exposure may intensify blood flow and influence the brain cooling processes, deteriorating cognitive functioning and mood state^20,21^.

Although a couple of recent systematic reviews and meta-analyses have summarized evidence on the associations between temperature and mental health for the general population^22,23^, such evidence summary has been lacking for children and adolescents. Given the value of early interventions in ameliorating later-life mental health endpoints, we aimed to conduct a systematic review and meta-analysis to quantify risks of mental health illness and suicide from high temperature extremes employing a structured population-exposure-comparator-outcome-study (PECOS) statement^24^. Our PECOS statement addressed the research question: ‘What is the effect of exposure to high temperature compared to low temperature exposure on the risks of mental health sequelae and suicide among children and adolescents?’

## Methods

### Eligibility criteria

A systematic review and meta-analysis was conducted following the 2020 Preferred Reporting Items for Systematic reviews and Meta-Analyses (PRISMA) guidelines^25^ (Supplementary File 1). The protocol for this work was registered on PROSPERO (registration ID: CRD42024604074). Studies were included for review if: (a) they were published in English peer-refereed journals, (b) the exposure included high temperature or ambient heat, (c) the outcome included mental health outcomes (e.g., anxiety, depression, suicides), and (d) the participants included children or adolescents aged ≤24 years. In this study, we have defined adolescents as individuals aged between 10 to 24 years to closely reflect the period of adolescent growth and transition to adulthood^26^. Studies were excluded if they were: (a) review articles, conference abstracts, news, or perspectives, or (b) did not report independent analysis conducted for children and adolescents (e.g., analysis for the general population aged between 10 and 80 years were excluded).

### Search strategy, selections and data collection

A systematic strategy was followed to search for articles published up to October 31, 2025 on PubMed, Web of Science, CINAHL Plus and PsycInfo. Specific search keywords associated with the outcome, exposure and target group were developed (Supplementary File 2). These search terms were initially created based on the PECOS statement with further modifications in reference to prior literatures on high temperature extreme and health^22,23^. The selection of databases was supported by prior review articles on climatic events and health^22,27^. The search terms were applied in the ‘title or abstract’ field without any filter. References of the pooled studies and climate-health-related reviews (Supplementary File 3) were also screened. A manual Google Scholar search was conducted to supplement.

After removing duplicated records, two researchers (KYL and CS) independently conducted screening based on title and abstract, followed by full-text evaluation. A third researcher (SB) was consulted about any conflict. We additionally reached out to the authoring teams of eligible studies to obtain further information, as required. Key characteristics from each included study were extracted and inserted in a pre-formatted summary table comprising study citation identifiers (author, publication year), study design, geographical setting, participant profile, analytic models, covariates, outcome, exposure and key findings with effect estimates.

### Risk of bias assessments

The quality of individual studies was evaluated by using the US National Toxicology Program’s Office of Health Assessment and Translation (OHAT) risk of bias rating tool^28^ (Supplementary File 4). Five dimensions of biases were assessed for each selected study. These included confounding bias, detection biases attributed to inadequate exposure characterization, and outcome assessments, reporting bias and other biases such as those related to study design, statistical method, non-adherence to protocol and others. Risk of bias was rated as ‘definitely high’, ‘probably high’, ‘probably low’, or ‘definitely low’ for each study. The risk of bias assessment was conducted independently by KYL and CS.

The overall confidence in the accumulative body of evidence on each exposure-outcome association was further evaluated using the Grading of Recommendations Assessment, Development and Evaluation (GRADE) framework within the OHAT approach^29^ (Supplementary File 5). Within this framework, an initial grade (high, moderate, low, or very low) was assigned for each exposure-outcome association based on four features, including exposure measurement prior to outcome development, use of individual-level data, use of comparison group, and inclusion of controlled exposure (not applicable in the present review)^30^. The grade is further adjusted based on eight dimensions. Five of these including risk of bias, indirectness, inconsistency, imprecision and publication bias shifted the grade of evidence downwards. The remaining three dimensions; namely, large magnitude of effect sizes, dose-response relationship, and confounding effect upgraded the overall quality of evidence. After initial rating and subsequent downgrading and upgrading, the overall confidence in the body of evidence was rated as ‘high’, ‘moderate’, ‘low’, or ‘very low’.

### Meta-analytic approach

Within the pooled studies, mental health outcomes and heat exposures were initially categorized based on similarity, and definitions of outcomes and exposures were constructed. Main outcomes were hospital visits or hospitalizations, as defined by International Classification of Diseases (ICD) for mental health disorders (F00-F99 in ICD-10 or 290.xx-319.xx in ICD-9), schizophrenia (F20-F29 in ICD-10 or 293.8, 295, 297, 298 in ICD-10-CM), depression (F32-F33 in ICD-10 or 296.2–296.39, 296.9–296.99, 300.4, 311 in ICD-9) and anxiety (F40-F41 in ICD-10 or 300–300.3, 300.5–300.9, 309.21, 309.81 in ICD-9). We further combined all sequelae (mental health disorders, schizophrenia, depression, anxiety) to define composite mental health illnesses. Suicide was defined as suicidal mortality based on ICD codes (X60-X84 in ICD-10 or Y87.0 in ICD-9 or E950.0–E958.9 in ICD-8). Analyses were conducted if three or more effect estimates derived from studies of comparable outcome and exposure were available.

Diversity of outcomes and heterogeneity in definitions of exposures to high outdoor temperature meant methodologically identical studies could be pooled together. This study employed the Hartung-Knapp-Sidik-Jonkman random-effects model for meta-analysis. This approach has been found to outperform the standard DerSimonian-Laird approach in terms of minimizing type 1 error (false positive findings) especially when the number of pooled studies is small^31^. For mental health outcomes, the association of exposure to high outdoor temperature relative to low (as defined in the respective study) with the risk of mental health sequelae was reported. Hence, estimates were first pooled using study-specific definitions of exposure to high outdoor temperature to measure their associations with hospital visits or hospitalizations from specific mental health disorders, schizophrenia, depression, and anxiety, relative to low outdoor temperature. Secondly, studies were also pooled to examine associations of study-specific definitions of exposure to high outdoor temperature with composite mental illnesses (including all sequelae).

For suicide, the effect estimates were reported with respect to unit increment in daily mean temperature. When effect estimates were provided for ΔT °C increments, they were standardized corresponding to 1 °C increment by dividing the effect estimates by the corresponding temperature increase (ΔT) in each study^23^.

In our main meta-analysis for mental health outcomes, if data were reported for different time intervals (lag periods), effect estimates corresponding to temperature measured on the day of hospitalization and diagnosis of the endpoint (lag 0) were used. Estimates were also pooled to calculate the effects of specific percentile-based definitions of high outdoor temperature (reported as ≥95^th^, ≥90^th^ and ≥75^th^ percentile of exposure distribution) upon mental health disorders, depression and composite mental illnesses (including all sequelae). To further study effects of cumulative exposure to high temperature, we conducted sub-group analysis pooling estimates by specific lag days of the day of hospital visit (lag03, lag05, lag06, lag07). We acknowledge that World Health Organization defines adolescents as individuals aged between 10–19 years^32^. As additional sensitivity test, we also conduced subgroup analysis for age groups 6–19 years to reflect children and adolescents, and 10–19 years to reflect only adolescents. We further conducted analysis by study design (time series and case-crossover). Between-study heterogeneity was examined using the *τ²* indicating the true heterogeneity between effect estimates and *I*^2^ statistics representing the proportion of variance attributable to heterogeneity rather than sampling error^33^ (with 25%, 50% and 75% being considered as low, moderate and high heterogeneity^34^). Publication bias was visually examined using funnel plots showing the standard error against the effect size and statistically tested using the Egger’s tests (when the number of pooled estimates is ≥10)^35,36^. As a sensitivity test, we repeated the main meta-analysis using the DerSimonian-Laird random-effects model.

Stata 18^37^ was utilized to perform all analyses in the present review. Relative risk (RR) and its 95% confidence interval (CI) were presented.

## Results

**Fig. 1:**
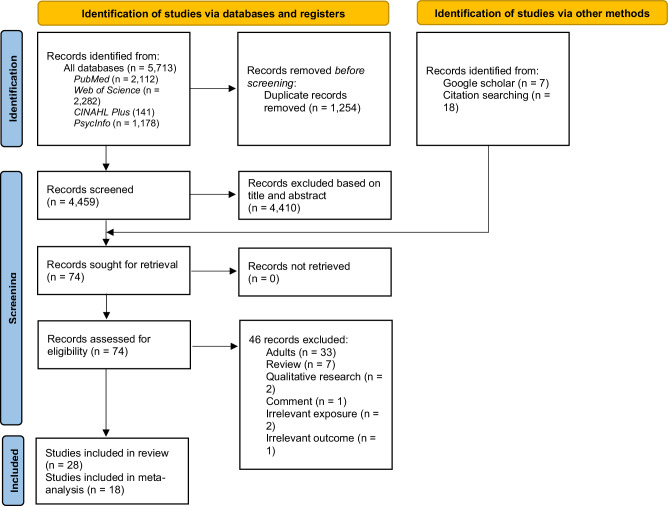
PRISMA flow diagram Preferred Reporting Items for Systematic Reviews and Meta-Analysis (PRISMA) flow diagram summarizing the search strategy and study selection process.

Our search identified 5713 records from databases and registers with a further 25 records identified from Google Scholar and reference lists of review articles. After screening, 74 full-text articles were retrieved for reviewing, of which 28 observational studies were eligible for systematic review. The PRISMA flowchart is presented in Fig. 1. The list of excluded records after full-text review with exclusion rationale and the list of included studies are presented in Supplementary Files 6 and 7, respectively. The authoring teams of two of the included studies^38,39^ were further contacted to obtain relevant additional information.

### Study profile

**Fig. 2:**
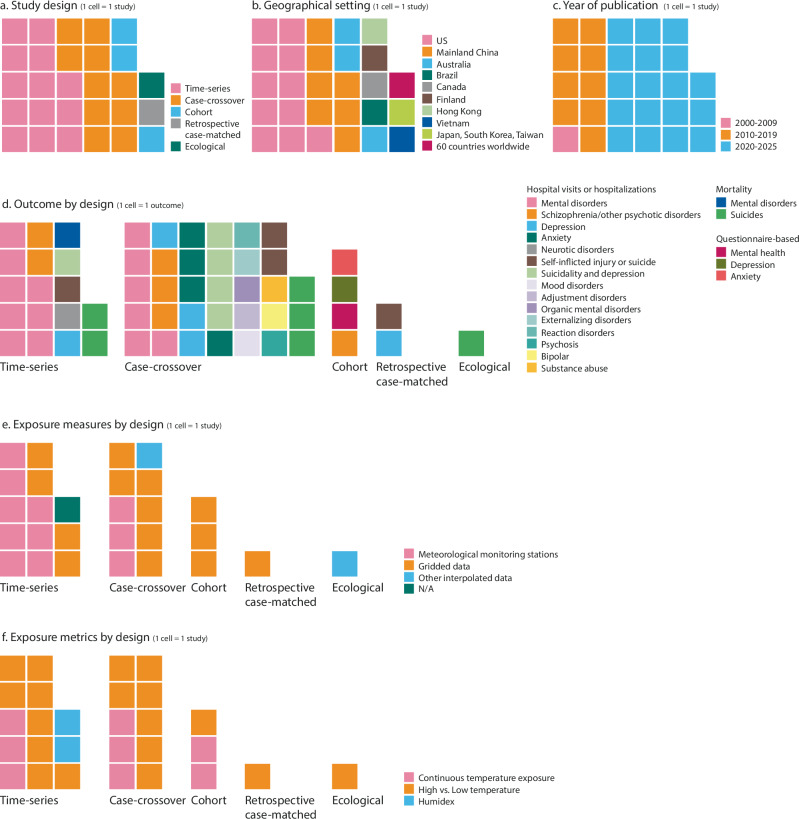
Descriptive characteristics. Waffle chart showing the key characteristics (including study design, geographical setting, year of publication, outcome, exposure measures and metrics) of the included studies.

Key characteristics of the 28 studies included in the systematic review (published over 2007-2025) are illustrated in the waffle charts (Fig. 2). These included 13 studies of time-series design (46%)^19,38,40–50^, 10 of case-crossover design (36%)^39,51–59^, three of cohort design (11%)^60–62^, one of retrospective matched-case design (4%)^63^, and one of ecological design (4%)^64^. Detailed study characteristics are summarized in Table 1 and presented in Supplementary File 8. Full study profile for individual studies is also shown in Supplementary File 9.

**Table 1:** Summary of study characteristics

Author	Year	Geographical Setting	Characteristics^a^	Outcome	Exposure
Time series design (*n* = 13)					
Basu et al.^38^	2018	California, US	Year of data: 2005-2013 Age: 6-18 Sample size: −28,592 (for mental health disorders) −25,128 (for neurotic disorders) −67,720 (for self-inflicted injury/suicide)	Emergency department visits for −mental health disorders (ICD-9: 290-319) −neurotic disorders (ICD-9 300–316) −self-inflicted injury/suicide (ICD-9: E950-E959)	Daily mean temperature at climate-zone level (*n* = 16) measured using data from meteorological monitoring stations weighted by distance from monitor and population during summertime (May-Oct). ➣Each 5.6°C (10 °F) increase
Bernstein et al^40^.	2022	US	Year of data: 2016-2018 Age: 0-18 Sample size: −69,995 (for mental health disorders), −33,229 (for suicidality and depression)	Emergency department visits for −mental health disorders (ICD-10CM: F00-F99) −suicidality and depression (ICD-10-CM: R45.85, R45.86, R45.87, R45.1, R45.4, R45.5, R45.6, F32, F33)	Daily maximum temperature at county-level measured using PRISM grid data (4-km) weighted by population during summertime (May-Sep). ➣Days of extreme heat (95^th^) as compared with minimum morbidity temperature
Chan et al^41^.	2018	Hong Kong	Year of data: 2002-2011 Age: <15 Sample size: 833	Mental health disorder hospitalizations (ICD-9: 290.xx-319.xx)	Daily mean temperature at city-level measured using data from a meteorological monitoring station. ➣28°C (4^th^ quartile) in reference to 19.4°C (lower quartile)
da Silva et al^42^.	2020	Curitiba City, Brazil	Year of data: 2010-2016 Age: 0-24 Sample size: 540	Mental health disorder hospitalizations (ICD-10: F00-F99)	Daily mean temperature at city-level measured using data from 3 meteorological monitoring stations. ➣Daily mean temperature in reference to 22.4°C
Isaksen et al^43^.	2016	King county, Washington, US	Year of data: 1980-2010 Age: 0-14 Sample size: −78,525 in 1980, 120,294 in 2010 (aged 0–4), −170,657 in 1980, 224,084 in 2010 (aged 5-14)	Mortality associated with mental health disorders (ICD-9: 290-316 & ICD-10: F01-F69)	Daily maximum temperature at county-level measured using PRISM grid data (1/16°) during summertime (May-Sep). ➣Heat day (99^th^) compared with a non-heat day
Mullins & White^19^	2019	California, US (for mental health); US (for suicide)	Year of data: 2005-2016 (for mental health), 1960-16 (for suicide) Age: 18-24 Sample size: 8294 (for mental health), 2,096,460 (for suicide)	Emergency department visits for mental health disorders (ICD-9-CM: 290-319; ICD-10: ‘F’ codes) Suicide (39-Cause code: 40; 34-Cause code: 350; ICD-7: 963, 970-979)	Daily mean temperature at county-level measured using PRISM grid (2.5 mile) weighted by inverse of square distance between the grid and population centroid of the county. ➣Each 1 °F increase
Nitschke et al^50^.	2007	Adelaide, Australia	Year of data: 1993-2006 Age: 0-14 Sample size: N/A	Hospital admission for mental diseases (ICD-9: 290-294-9, 580-5999; ICD-10: N00-N39)	Daily maximum temperature during spring and summer. ➣≥35°C for ≥3 consecutive days (heatwave episodes)
Niu et al^44^.	2020	Beijing, China	Year of data: 2016-2018 Age: <18 Sample size: 1653	Emergency admissions associated with mental health disorders (ICD-10: F00-F99)	Daily mean temperature at city-level measured using data from 3 meteorological monitoring stations weighted by water vapor pressure and wind velocity. ➣High temperature (9.2°C; 90^th^) in reference to -2.4°C
Parks et al^45^.	2020	US	Year of data: 1980-2017 Age: 5-14 Sample size: 10,719	Suicide (ICD-9: E950-E959 & ICD-10: X60-X84)	Monthly temperature at state-level using ERA5 grid data (30-km) weighted by population. ➣Each 1.5°C increase
Trang et al^49^.	2016	Hanoi, Vietnam	Year of data: 2008-2012 Age: <18 Sample size: 655	Hospital admission for mental health disorder (ICD-10: F00-F99, except F60-F69)	Daily maximum temperature at the city-level using data from meteorological stations. ➣35°C for one day or≥3 or ≥7 consecutive days
Wang et al^47^.	2018	Hefei, China	Year of data: 2005-2014 Age: ≤20 Sample size: 3361	Hospital admission for schizophrenia (ICD-10: F20-F29)	Daily mean temperature measured using data from meteorological station during summertime time (May-Oct). ➣28°C (75^th^) in reference to lower temperature at 24.8°C (50^th^)
Zhou et al^46^.	2023	Chongqing, China	Year of data: 2014-2019 Age: ≤18 Sample size: 13,329	Outpatient visits for depression (ICD-10: F32)	Temperature at city-level measured using data from a meteorological monitoring station. ➣Extremely high humidex ( > 40)
Zhou et al^48^.	2024	Liuzhou, China	Year of data: 2013-2020 Age: ≤20 Sample size: 966	Hospital admission for schizophrenia (ICD-10: F20-F20.9)	Daily mean temperature measured using data from meteorological station. ➣High temperature in reference to 21.7°C (75^th^)
Case-crossover design (*n* = 10)					
Cohen et al^55^.	2024	New York, US	Year of data: 1995-2014 Age: ≤24 Sample size: −811,439 (for mood disorders), −447,286 (for anxiety disorders), −134,839 (adjustments disorders), −27,888 (for schizophrenia and other psychotic disorders)	Hospital visits for −mood disorders (ICD-9-CM: 293.83, 296, 300.4, 311.0) −anxiety disorders (293.84, 300, 300.10, 300.2, 300.3, 300.5, 300.89, 300.9, 308.0, 308.1-308.4, 308.9, 309.81, 313.0-313.1, 313.21-313.22, 313.3, 313.82, 313.83) −adjustments disorders (309.0, 309.1, 309.22-309.24, 309.28, 309.29, 309.3, 309.4, 309.82, 309.83, 309.89, 309.9) −schizophrenia and other psychotic disorders (293.8, 295, 297, 298)	Daily temperature range (difference between maximum and minimum temperatures) at ZIP code level measured using NLDAS-2 Forcing grid data (0.125° or 11-km*14 km) weighted by population. ➣Higher daily temperature range (5.2°C) in reference to minimum daily temperature range (0.1°C)
He et al^56^.	2025	New South Wales, Australia	Year of data: 2001-2020 Age: 0-18 Sample size: −91,938 (emergency department visits; mental disorder) −36,241 (emergency department visits; suicidality and depression) −30,017 (hospital admissions; mental disorder) −8,114 (hospital admission; suicidality and depression)	Emergency department visits for: −mental disorders (ICD-10-AM: F00-F99) −suicidality and depression (ICD-10-AM: R45.85, R45.86, R45.87, R45.1, R45.4, R45.5, R45.6, F32, F33) Hospital admission for: −mental disorders (ICD-10-AM: F00-F99) −suicidality and depression (ICD-10-AM: R45.85, R45.86, R45.87, R45.1, R45.4, R45.5, R45.6, F32, F33)	Temperature using the ERA5 data (0.25°) ➣Heatwave days in reference to non-heatwave days
Kim et al^51^.	2016	Korea, Japan, Taiwan	Year of data: 1992-2010 (Korea), 1972-2010 (Japan), 1994-2007 (Taiwan) Age: 10-24 Sample size: 21,061	Suicide (ICD-8 & ICD-9: E950.0-E958.9; ICD-10: X60-X84)	Daily mean temperature at city-level measured using data from meteorological monitoring stations. ➣Location-specific °C increase
Ndovu et al^57^.	2025	California, US	Year of data: 2005-2019 Age: <19Sample size: −512,977 (mental disorders) −163,187 (suicidality and depression)	Emergency department visits and any unscheduled hospital admissions for: −mental health disorders (ICD-9: 290-319; ICD-10: F00-F99) −suicidality and depression (ICD-9: 296.2-296.3, 311, V62.84; ICD-10: R45.8, F32-F33, X60-X84)	Daily maximum temperature at ZIP-code-level measured using the PRISM grid data (4-Km) ➣Extreme heat events (95^th^ percentile)
Niu et al^52^.	2023	New York City, US	Year of data: 2005-2011 Age: 6-17 Sample size: −82,982 (for mental health disorders), −8238 (for anxiety), −13,060 (for depression), −4301 (for suicide and self-inflicted injury), −7696 (for externalizing disorders), −5575 (for reaction disorders), −13,742 (for psychosis), −6803 (for bipolar) −16,907 (for substance abuse)	Emergency department visits and hospitalizations for −mental health disorders (ICD-9: 290-299) −Anxiety (ICD-9: 300-300.3, 300.5-300.9, 309.21, 309.81) −Depression (ICD-9: 296.2-296.39, 311, 300.4, 296.9-296.99) −Suicide and self-inflicted injury (ICD-9: E95) −Externalizing disorders (ICD-9: 312-313.82) −Reaction disorders (ICD-9: 308-309.2, 309.22-309.8, 309.82-309.9) −Psychosis (ICD-9: 290–295.95, 297–298.9) −Bipolar disorder (ICD-9: 296-296.16, 296.4-296.89) −Substance abuse (ICD-9: 303-305.93)	Daily minimum temperature at city-level measured using data from 4 meteorological monitoring stations during summertime (Jun-Aug). ➣95^th^ percentile in reference to minimum risk temperature
Rahman et al^53^.	2023	California, US	Year of data: 2014-2019 Age: ≤24 Sample size: 2875	Suicide (ICD-10: X60-X84, Y87.0)	Daily min and max temperatures at census tract level measured using gridMET data (4-km). ➣Each 1°C increase
Stowell et al^59^.	2023	US	Year of data: 2016-2019 Age: 0–17 Sample size: −26,366 (mental, behavioral disroders) −16,579 (suicidality and depression)	Emergency department visits for −mental, behavioral disroders (ICD-10: F00-F99) −suicidality and depression (ICD-10: R45.85-R45.87, R45.1, R45.4-R45.6, F32, F33)	Daily maximum temperature at county-level measured using PRISM grid data (4-km) weighted by population during summertime (May-Sep). ➣Extreme heat (95^th^) in reference to 50^th^ percentile
Villeneuve et al^39^.	2023	Canada	Year of data: 2002-2015 Age: <25 Sample size: 6813	Suicide (ICD-10: X60-X84)	Daily mean temperature at postal code level measured using interpolated metrics weighted by daily count of suicides deaths. ➣Each 9.6°C increase
Zhang et al^54^.	2020	Shenzhen, Zhaoqing and Huizhou, China	Year of data: 2016-2018 (Shenzhen, Zhaoqing), 2013-18 (Huizhou) Age: <18 Sample size: 55,934	Hospital outpatient visits for depressive disorders (ICD-10: F32-F33), anxiety (F40-F41), organic mental health disorders (F00-F09), schizophrenia (F20-F29), & affective disorders (excluding F30-F31 & F34-F39).	Temperature using data from meteorological monitoring stations. ➣30°C (97.5^th^) in reference to minimum risk temperature (18°C)
Zhong et al^58^.	2025	Anhui, China	Year of data: 2019-2021 Age: <18 Sample size: 76,023	Outpatient visits for: −mental and behavioral disorders (ICD-10: F00-F99) −schizophrenia (ICD-10: F20-F21) −depression (ICD-10: F32-F33) −anxiety (ICD-10: F40-F41)	Daily mean temperature using the CN05.1 grid data (0.25°). ➣Heat exposure (both daily max and min temperature ≥90^th^ percentile for ≥1 day)
Cohort design (*n* = 3)					
Hu et al^62^.	2025	China	Year of data: 2021 Age: 10-18 Sample size: 19,852	−Depression measured based on Patient Health Questionnaire-9 (PHQ-9) and defined using the threshold of 10 −Anxiety measured using the 7-item Generalized Anxiety Disorder (GAD-7) and defined using the threshold of 10	Temperature using the ERA5-Land dataset (0.1°) ➣Each additional heatwave increase
Komulainen et al^60^.	2022	Finland	Year of data: 1990-2017 Age: 10 Sample size: 365,482	Hospital admission (ICD-10: F20) or emergency unit visits or receiving outpatient care for schizophrenia	Daily mean temperature from birth to 10th birthday at residential zip code level measured using gridded data (10 km). ➣Highest quintile (quintile 5) in reference to the lowest quintile
Xu et al^61^.	2018	Australia	Year of data: 2008-2014 Age: 6-11 Sample size: 14,096	Mental health based on the Strengths and Difficulties Questionnaire (SDQ).	Yearly average of maximum temperature at postcode level measured using gridded data (1 km). ➣Each 1 °C increase
Retrospective matched-case design (*n* = 1)					
Runkle et al^63^.	2025	North Carolina, US	Year of data: 2008-2021 Age: 5-24 Sample size: −1,029 (major depressive disorder) −3,712 (suicidal behavior)	Emergency department visits for −major depressive disorder (ICD-9: 296.3; ICD-10: F33) −suicidal behavior (ICD-9: E95, V628.4; ICD-10: X60-X84, R45.851, T14.91)	Daily mean temperature at county-level measured using the nClimGrid product (0.0417°). ➣Heatwave days in reference to non-heatwave days
Ecological design (*n* = 1)					
Ngu et al^64^.	2017	Sixty countries (excluding US, China, India and Russia)	Year of data: 1979-2016 Age: 5-24 Sample size (countries): −female: 16 (aged 5-14), 9 (aged 15-24) −male: 10 (aged 5-14), 8 (aged 15-24)	Suicide defined as fatal intentional self-harm derived from the WHO’s mortality database.	Temperature at country-level measured using ERA5 data. ➣One unit increase in heatwave count

N/A indicates that information was not reported in the article.

*ERA5* fifth generation ECMWF atmospheric reanalysis of the global climate, *ICD* International Classification of Diseases, *NLDAS* North American Land Data Assimilation System, *PRISM* Parameter-elevation Relationships on Independent Slopes Model.

^a^The sample size was calculated from the reported population size and % of events among individuals aged ≤24 years for Basu et al., 2018, da Silva et al., 2020, Kim et al., 2016 and Zhang et al., 2020.

➣indicates the article’s key metrics.

### Risk of bias assessments

Application of the OHAT risk of bias rating tool found that risk of biases associated with biased reporting and other biases were probably low (Supplementary File 10; see Supplementary File 11 for assessments of individuals studies). Confounding bias was judged to be probably high in two included studies of time-series^45^ and ecological design^64^ which did not adjust for any potential covariates. Detection bias associated with exposure assessments was judged as probably high in 10 studies [with seven studies of time-series design (54%)^41,42,44,46–48,50^ and three studies of case-crossover design (43%)^51,52,54^] due to temperature assessments based on a single or very few meteorological monitoring stations for the entire study area and resulting poor resolution. Detection bias associated with outcome assessments was evaluated as probably high in one study^50^ due to inclusion of ICD codes unrelated to mental health.

The overall confidence in the body of evidence was evaluated based on the GRADE guideline (Supplementary File 12). The risk of bias constituted a downgrading element for the association between high temperature exposure and mental disorders, with 39% of studies measuring the exposure at sparse resolution directly from a few meteorological monitoring stations, and one study employed imprecise outcome measurements, which subsequently shifted the initial grade of confidence in the body of evidence downwards from ‘moderate’ to ‘low’. The final grade was adjusted from ‘moderate’ to ‘low’ for anxiety owing to inconsistency of evidence and imprecision (wider confidence intervals), as well as depression due to inconsistency of evidence. The final grade of confidence in the body of evidence remained moderate for schizophrenia and suicide. For suicide, inconsistency of evidence constituted a downgrading element, while dose-response was an upgrading element, so that the overall grade remained moderate.

### Meta-analysis

**Fig. 3:**
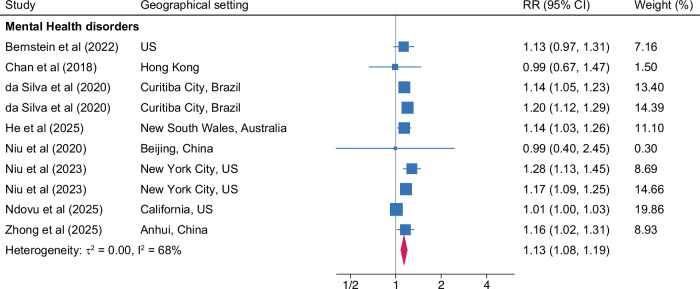
Forest plot of meta-analysis showing associations between exposure to high temperature and mental health disorders. The plot depicts associations of exposure to high temperature on hospital visits or hospitalizations for mental health disorders compared with low temperature exposure. The blue square indicates the effect size with 95% confidence intervals (CI) in horizontal line for individual studies. The mid-point of the pink diamond on the x-axis represents the overall effect size, whereas the width of the diamond represents the 95% CI. The gray solid line represents 1. *Note*. *Mental health disorders* is defined based on ICD-10: F00-F99 or ICD-9: 290.xx-319.xx.

**Fig. 4:**
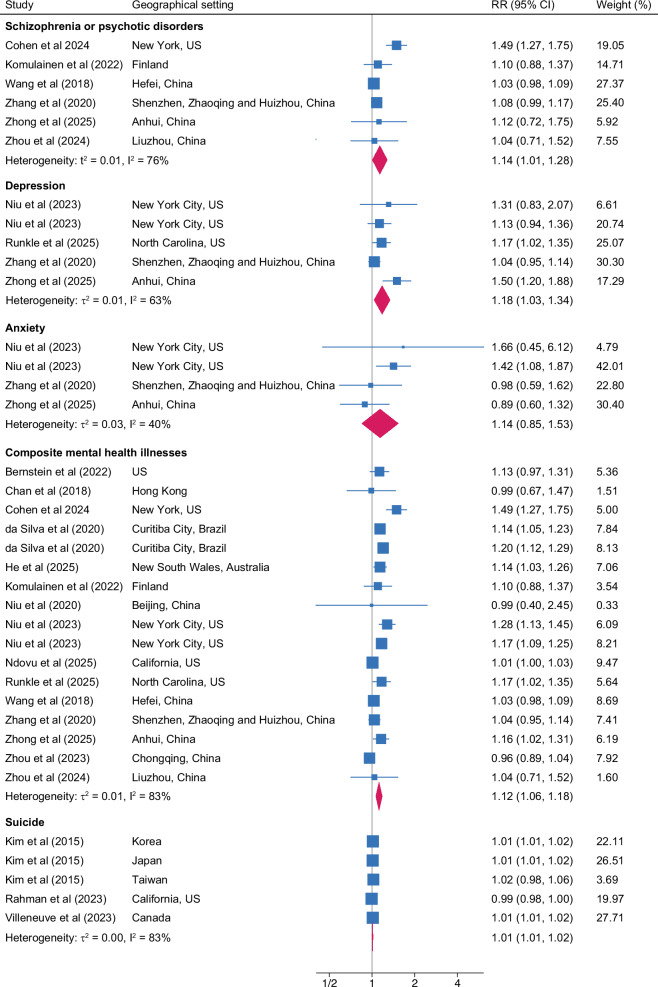
Forest plot of meta-analysis showing associations between exposure to high temperature and mental health sequelae. The plot depicts associations of exposure to high temperature on hospital visits or hospitalizations for schizophrenia or psychotic disorders, depression, anxiety, composite mental health illnesses (all sequelae) compared with low temperature exposure. The plot for suicide depicts the association with each 1 °C increase in daily mean temperature. The blue square indicates the effect size with 95% confidence intervals (CI) in horizontal line for individual studies. The mid-point of the pink diamond on the x-axis represents the overall effect size, whereas the width of the diamond represents the 95% CI. The gray solid line represents 1. *Note*. 1. *Mental health disorders* is defined based on ICD-10: F00-F99 or ICD-9: 290.xx-319.xx. 2. *Schizophrenia or psychotic disorders* is defined based on ICD-10: F20-F29 or ICD-10-CM: 293.8, 295, 297, 298. 3. *Depression* is defined based on ICD-10: F32-F33 or ICD-9: 296.2-296.39, 311, 300.4, 296.9-296.99. 4. *Anxiety* is defined based on ICD-10: F40-F41 or ICD-9: 300-300.3, 300.5-300.9, 309.21, 309.81. 5. *Composite mental illnesses* comprised all sequelae including mental health disorders, schizophrenia or psychotic disorders, depression and anxiety. 6. *Suicide* was defined from ICD codes (ICD-10: X60-X84, Y87.0; ICD-9 and ICD8: E950.0-E958.9). 7. Niu et al (2023) provided estimates for two age subgroups including 6-11, and 12-17.

Diversity in definitions of outcomes and variables meant we could include 18 methodologically comparable studies for meta-analysis. Meta-analyses conducted by specific mental health sequelae are presented in Table 2 and Figs. 3 to 4. Relative to low outdoor temperature, exposure to high temperature was associated with 13% higher risk of hospital visits or hospitalizations for mental health disorders (RR, 1.13; 95% CI, 1.08 to 1.19; *n* = 720,512), 14% higher risk for schizophrenia or other psychotic disorders (RR, 1.14; 95% CI, 1.01 to 1.28; *n* = 529,654), and 18% higher risk for depression (RR, 1.18; 95% CI, 1.03 to 1.34; *n* = 146,046). Consistent association was observed for anxiety, although the results remained insignificant. The heterogeneity remained moderate-to-high for mental health disorders (*I*^2^ = 68%), schizophrenia (*I*^2^ = 76%), and depression (*I*^2^ = 63%), whereas it was moderate for anxiety (*I*^2^ = 40%). Overall, exposure to high temperature was associated with a higher risk of hospital visits or hospitalizations for composite mental illnesses including all sequelae (RR, 1.12; 95% CI, 1.06 to 1.18, *I*^2^ = 83%; *n* = 1,214,867) relative to low temperature. Every 1 °C increase in temperature was associated with an estimated 1.0% increment in suicide (RR, 1.01; 95% CI, 1.00 to 1.02; *n* = 30,749), with a high level of heterogeneity (*I*^2^ = 83%).

**Table 2:** Meta-analysis of associations of exposure to high temperature with hospital visits or hospitalizations for mental health sequelae among children and adolescents relative to low temperature

Outcome	Estimate count [study count]	Relative risk (95% CI)	*τ²*	*I*^2^ (%)
**Mental health sequelae**				
Mental health disorders^a^	10 [8]	1.13 (1.08, 1.19)	0.00	68
Schizophrenia or other psychotic disorders^b^	6 [6]	1.14 (1.01, 1.28)	0.01	76
Depression^c^	5 [4]	1.18 (1.03, 1.34)	0.01	63
Anxiety^d^	4 [3]	1.14 (0.85, 1.53)	0.03	40
Composite mental illnesses (all sequelae)^e^	17 [15]	1.12 (1.06, 1.18)	0.01	83
Suicide	5 [5]	1.01 [1.00, 1.02]	0.00	83
Percentile-based specific definitions of high temperature extreme				
≥95^th^ pc (versus study-specific lower temp.)				
Mental health disorders	4 [3]	1.13 (1.02, 1.25)	0.01	86
Depression	4 [3]	1.11 (0.99, 1.24)	0.00	27
Composite mental illnesses (all sequelae)	5 [4]	1.11 (1.02, 1.21)	0.01	84
≥90^th^ pc (versus study-specific lower temp.)				
Mental health disorders	6 [5]	1.13 (1.05, 1.22)	0.00	73
Depression	5 [4]	1.19 (1.04, 1.37)	0.01	55
Composite mental illnesses	7 [6]	1.11 (1.04, 1.19)	0.00	74
≥75^th^ pc (versus study-specific lower temp.)				
Mental health disorders	7 [6]	1.12 (1.04, 1.21)	0.00	69
Composite mental illnesses (all sequelae)	9 [8]	1.09 (1.03, 1.16)	0.00	77
Cumulative effects over lag periods (composite mental health illnesses)				
lag03	4 [4]	1.12 (0.99, 1.25)	0.01	78
lag05	6 [5]	1.13 (1.00, 1.29)	0.02	84
lag06	3 [3]	1.28 (1.00, 1.64)	0.03	77
lag07	4 [4]	1.13 (0.97, 1.32)	0.01	56
Age groups (composite mental health illnesses)				
6–19 years	3 [3]	1.19 (1.04, 1.36)	0.00	31
10–19 years	3 [3]	1.17 (1.11, 1.24)	0.00	1
Study design (composite mental health illnesses)				
Time-series	8 [7]	1.08 (1.01, 1.15)	0.00	59
Case-crossover	8 [7]	1.14 (1.04, 1.25)	0.01	91

*CI* confidence intervals, *CM* clinical modification, *ICD* International Classification of Diseases, *pc* percentile, *temp.* temperature.

^a^ICD-10: F00-F99 or ICD-9: 290.xx-319.xx.

^b^ICD-10: F20-F29 or ICD-10-CM: 293.8, 295, 297, 298.

^c^ICD-10: F32-F33 or ICD-9: 296.2-296.39, 296.9-296.99, 300.4, 311.

^d^ICD-10: F40-F41 or ICD-9: 300-300.3, 300.5-300.9, 309.21, 309.81.

^e^The definition of composite mental illnesses comprised all sequelae including mental health disorders, schizophrenia or psychotic disorders, depression and anxiety.

Meta-analysis using specific definitions of high outdoor temperature reported consistent findings. Exposure to high outdoor temperature defined as exposure to temperature ≥95^th^ percentile of the distribution was associated with 13% higher risk for mental health disorders (RR, 1.13; 95% CI,1.02 to 1.25), 11% higher risk for depression (RR, 1.11; 95% CI, 0.99 to 1.24) and 11% higher risk for composite mental illnesses including all sequelae (RR, 1.11; 95% CI, 1.02 to 1.21), relative to low temperature. Other definitions of high outdoor temperature extremes, such as temperature being ≥90^th^ and ≥75^th^ percentile distribution consistently showed higher risks of mental health disorders and composite mental illnesses.

Further analysis estimating the cumulative effects found that high outdoor temperature exposure relative to low temperature measured over lag 0–5 days and lag 0–6 days were associated with 13% (RR, 1.13, 95% CI, 1.00 to 1.29) and 28% (RR, 1.28, 95% CI, 1.00 to 1.64) higher risks of composite mental health illnesses, respectively. Subgroup analysis by age groups found that high outdoor temperature exposure relative to low temperature was associated with 19% (RR, 1.19, 95% CI, 1.04 to 1.36) higher risk of composite mental health illnesses for individuals aged 6–19 years and 17% (RR, 1.17, 95% CI, 1.11 to 1.24) higher risk for those aged 10–19 years. Additional subgroup analysis by study design also showed that exposure to high temperature was associated with higher risks of hospital visits or hospitalizations for composite mental health illnesses for both time-series (RR, 1.08; 95% CI, 1.01 to 1.15) and case-crossover (RR, 1.14; 95% CI, 1.04 to 1.25) study designs. Our sensitivity analysis using the DerSimonian-Laird random-effects model produced comparable pooled effect estimates as in the main meta-analysis (Supplementary File 13).

Funnel plots are shown in Supplementary File 14. As shown from the funnel plots for mental health disorders and composite mental health illnesses, there was a suggestion of missing studies on the top left-hand side and bottom of the plots, plausibly indicating potential publication bias. However, the non-significant results from the Egger’s tests for mental health disorders (*p* = 0.652) and composite mental health illnesses (*p* = 0.603) likely suggested that such asymmetry in the funnel plots were not pronounced in the included studies in this meta-analysis.

## Discussion

To our knowledge, this systematic review and meta-analysis was the first to comprehensively synthesize accumulated evidence on the associations between heat exposure and mental health among children and adolescents. We found that exposure to high temperature was associated with 13% higher risks of hospital visits or hospitalization for mental health disorders, 14% for schizophrenia, 18% for depression and 12% for composite mental health illnesses (all sequelae), relative to low temperature. Analysis with specific definitions of high temperature extremes such as exposures to ≥95^th^, ≥90^th^ and ≥75^th^ percentiles of exposure distribution produced consistent results. We also found that each 1 °C increment in temperature exposure was associated with 1% higher risk of suicidal mortality. The overall confidence in the body of evidence as per the GRADE guideline was rated as ‘moderate’ for the associations between heat extremes and schizophrenia/other psychotic disorders and suicide, and ‘low’ for mental health disorders, depression and anxiety. There was moderate to high heterogeneity (in terms of *I*^2^) across the mental health outcomes. These evidences of positive association between exposure to high temperature and mental health corroborate those reported in previous reviews among adolescents^65,66^ and meta-analyses within a general population^22,23^.

Heterogeneity in the definitions of exposure to high temperature meant we could examine the overall association of exposure to high temperature relative to low with risks of mental health sequelae as defined in respective studies. Absolute temperature, expressed as unit degree centigrade increment in daily temperature, was used for suicide. Overall, 10 of 28 (36%) included studies reported high detection bias. This stems from the inability to accurately measure spatial and temporal variations in high temperature exposures at appropriate resolutions, with potential for exposure misclassification and associated positive or negative biases. These studies measured temperature at low spatial resolution using data from a few meteorological monitoring stations to represent exposures across spatially heterogeneous study areas. Relatively refined gridded estimates of temperature to capture exposures were used in 14 studies. Majority of the studies measured temperature on the same day of the reporting of the mental health event (lag 0). Few studies considered lag structures of short periods up to 7 or 14 days for capturing short-term effects on acute mental health outcomes. Future studies should also examine the associations at varying lag structures including long-term temperature measurements (monthly and annual temperature assessments) to understand accumulated effects.

Differential adjustments for confounding in the pooled estimates may also explain the observed heterogeneity in *I*^2^. Approximately half of the studies were of time series design and as such could not adjust for individual-level confounding variables including individual socio-demographics, pre-existing health status etc^67^. Strategies such as adding a variable that splits the data study period into different intervals (such as month), fitting a periodic function, and fitting a spline function are generally employed to address this problem^68^. Nine out of 13 time-series studies included fitted a spline function, one adjusted for day of study and day of year, another study adjusted for county-by-month and county/state-by-year fixed effects to account for long-term and seasonal trends. One study employed Bayesian spatio-temporal model, while another excluded autumn and winter to account for seasonality. The 10 case-crossover studies employed self-matching wherein a case serving as its own control at a different time, thus being able to account for time in-varying individual confounders and long-term trends by-design. Although well-suited for recurrent exposures (with day-to-day variations) and acute outcomes, this design is unable to account for time-varying factors and the influence of chronic exposures^69^. One of the cohort studies with hospital record linkage systematically collect data on large populations, thereby prospectively linking chronic health outcomes, as they accumulate in response to specific exposure over time.

Our study has several strengths. This is the first study to systematically synthesize evidence on the association between heat exposure and mental health in children and adolescents. We employed rigorous and replicable protocols throughout including PRISMA checklist for reporting, OHAT risk of bias assessment within the GRADE framework, systematic examination of heterogeneity and effects across vulnerable subgroups for a range of mental health outcomes. Secondly, we employed the *Hartung-Knapp-Sidik-Jonkman random-effects model* for metanalysis which has been known to outperform the traditional *DerSimonian–Laird random-effects model*, providing more robust (low type 1 error) results when the number of individual studies are small. Lastly, twenty-six of the 28 included studies (93%) were conducted in either high-income (18/28; 65%) or upper-middle-income (8/28; 26%) economies. With the increasing severity and frequency of heat extremes, these findings have important policy implications for high- and upper-middle-income settings, especially providing timely opportunities for optimizing multilevel policies and interventions to protect against adverse health effects in children and adolescents. This will entail effective synchronization of national-level mitigation policies^70^ for multi-sectoral decarbonization with local interventions to strengthen community resilience^71^, supplemented with individual-level adaptation strategies. Interventions such as retrofitting cooling infrastructures within school settings, subsidizing air-conditioning costs, allocating community healthcare resources, provisioning green buildings to ameliorate heat island effects in cities and heat action planning constitute key strategies to strengthen community resilience. The importance of community-based heat adaptation behavioral interventions in improving heat literacy and behavioral adaptation, and heat-related health outcomes has been acknowledged^72^. Pediatricians and schools can play a crucial role in devising educational plans to inform children and adolescents as well as their guardians the importance of minimizing heat exposures as individual lifestyle choice^73,74^.

Among the limitations, we relied on a limited number of accumulated evidence for the systematic review and estimates were pooled based on categorizations of heat exposures and mental health across consistently comparable studies. As such, caution has to be taken when interpreting the pooled results. Available data was sparse to enable examination of specific dose-response relationships for mental health outcomes. Secondly, our pooled results are unlikely to be generalizable due to under-representation of population from lower-income economies (likely to be more prone to extreme temperature exposure). Thirdly, the inclusion of children and adolescents aged 24 years or below also implied that our findings are not generalizable to adults and older persons. Fourthly, heterogeneity was reported to be moderate-to-high in meta-analyses. Fifth, a small number of studies of variable designs (time series, case-crossover and cohort studies) meant we did not have sufficiently powered data to investigate effects by sub-groups and their interactions, such as age groups (younger children adolescents), ethnicity and socio-economic status. Future studies may consider case-crossover design nested within a cohort, given their potential to overcome confounding bias and address interactions between heat exposures, socio-demographics and phenotypes^69^. Lastly, 68% of the pooled studies (19/28) measured hospital visits or hospitalizations for mental health disorders. Ascertainment of mental health disorders from hospital records is associated with under-reporting of less severe cases and potential underestimation. Surveillance of mental disorders among children and adolescents heavily relies on caregivers’ recall of diagnosis, initiatives of reporting, interpretations and potential stigma associated with cultural norms related to help-seeking behaviours for mental health difficulties^75^. Previous studies have reported under-reporting of adolescent mental health disorders by ethnicity^76^ and socio-economic deprivation^77^.

In this systematic review and meta-analysis, we identified positive associations between heat exposure and risks of mental health sequelae and suicide. These findings suggest the need for early life interventions and evidence-based public health policies for preventing climate-induced mental illnesses and developing adaptation strategies among younger population^73^. Global scale climatic models predict increasing temperature variability during warm seasons in low-income tropical countries including Amazonia and Southern African nations, Sahel, India and Southeast Asia^78^. These countries not only have low gross domestic product with limited capacities in terms of financial and healthcare resources but also have relatively higher birth rates as compared to countries of developed economies, with more children and adolescents at-risk. Future research needs to be focused on these countries of low resource settings to understand the joint effects of poverty and increasing climatic variability on mental health in early life. Furthermore, at a national level, exposure to climate extremes including heat extremes is socio-spatially patterned^79^ and expected to exacerbate within-country health disparities^80^ among children and adolescents, amplify vulnerabilities, and potentially create intergenerational injustice. Governments should pool resources and incentivize financial and technical investments for developing comprehensive national-level heat action plans as a part of national capacity development. This should essentially leverage on local meteorological, sociodemographic and health datasets to create efficient systems for early warning and identification of at-risk population and areas for timely interventions^81^. Future studies should also discern dose-response relationship between heat exposure and mental health as well as the interaction effects with socio-demographic factors in early life.

## Supplementary information

Supplementary Information

## Data Availability

For data availability of individual studies, please refer to the relevant publication.

## Code availability

No custom code was used in this study.
