# Supplementary material for: Associations of heat exposure with mental health and suicide in children and adolescents: a systematic review and meta-analysis
Source: Npj Ment Health Res. 2026 Jan 30;5:7. doi: 10.1038/s44184-026-00190-w (PMC12859151; doi:10.1038/s44184-026-00190-w)
Supplement: Supplementary file 1 — Supplementary Information [file 44184_2026_190_MOESM1_ESM.pdf]

Supplementary information

Associations of heat exposure with mental health and suicide in children and adolescents: a systematic review and meta-analysis

Ka Yan Lai[1,2,3*], Sarah Bauermeister[4], Chinmoy Sarkar[1,2,3,4]

[1]Healthy High Density Cities Lab, HKUrbanLab, The University of Hong Kong, Hong Kong Special Administrative Region, China
[2]Department of Urban Planning & Design, Faculty of Architecture, The University of Hong Kong, Hong Kong Special Administrative Region, China
[3]Institute for Climate and Carbon Neutrality, The University of Hong Kong, Hong Kong Special Administrative Region, China
[4]Department of Psychiatry, University of Oxford, Oxford, United Kingdom

*Correspondence: Dr Ka Yan Lai | kyylai@hku.hk
Healthy High Density Cities Lab, HKUrbanLab, The University of Hong Kong, Knowles Building, Pokfulam Road, Pokfulam, Hong Kong Special Administrative Region, China.

Table of Content

	Content	Page no.
Supplementary File 1	The PRISMA Statement	3
Supplementary File 2	Search results for PubMed, Web of Science, CINAHL Plus, and PsycInfo	6
Supplementary File 3	List of review articles	8
Supplementary File 4	Office of Health Assessment and Translation (OHAT) risk of bias rating tool	9
Supplementary File 5	Grading of Recommendations Assessment, Development and Evaluation (GRADE) guideline	14
Supplementary File 6	List of excluded articles after full-text review	16
Supplementary File 7	List of included studies for systematic review	20
Supplementary File 8	Systematic review	22
Supplementary File 9	Study characteristics of individual studies	27
Supplementary File 10	Summary of risk of bias of individual studies as per the US National Toxicology Program's Office of Health Assessment and Translation (OHAT) risk of bias rating tool	56
Supplementary File 11	Risk of bias summary for individual students	58
Supplementary File 12	Assessments of confidence in the body of evidence as per the Grading of Recommendations Assessment, Development and Evaluation (GRADE) guideline.	86
Supplementary File 13	Sensitivity analysis using the DerSimonian-Laird random-effects model	88

Supplementary File 14	Funnel plots examining biases.	89
	References	90

Supplementary File 1. The PRISMA Statement.

Section and Topic	Item #	Checklist item	Location where item is reported
**TITLE**			
Title	1	Identify the report as a systematic review.	p. 1
**ABSTRACT**			
Abstract	2	See the PRISMA 2020 for Abstracts checklist.	p. 2
**INTRODUCTION**			
Rationale	3	Describe the rationale for the review in the context of existing knowledge.	p.3-4
Objectives	4	Provide an explicit statement of the objective(s) or question(s) the review addresses.	p. 4
**METHODS**			
Eligibility criteria	5	Specify the inclusion and exclusion criteria for the review and how studies were grouped for the syntheses.	p. 4
Information sources	6	Specify all databases, registers, websites, organisations, reference lists and other sources searched or consulted to identify studies. Specify the date when each source was last searched or consulted.	p. 4
Search strategy	7	Present the full search strategies for all databases, registers and websites, including any filters and limits used.	p. 4, Supplementary Files 2 and 3
Selection process	8	Specify the methods used to decide whether a study met the inclusion criteria of the review, including how many reviewers screened each record and each report retrieved, whether they worked independently, and if applicable, details of automation tools used in the process.	p. 4-5
Data collection process	9	Specify the methods used to collect data from reports, including how many reviewers collected data from each report, whether they worked independently, any processes for obtaining or confirming data from study investigators, and if applicable, details of automation tools used in the process.	p. 4-5
Data items	10a	List and define all outcomes for which data were sought. Specify whether all results that were compatible with each outcome domain in each study were sought (e.g. for all measures, time points, analyses), and if not, the methods used to decide which results to collect.	p. 4
	10b	List and define all other variables for which data were sought (e.g. participant and intervention characteristics, funding sources). Describe any assumptions made about any missing or unclear information.	p. 5
Study risk of bias assessment	11	Specify the methods used to assess risk of bias in the included studies, including details of the tool(s) used, how many reviewers assessed each study and whether they worked independently, and if applicable, details of automation tools used in the process.	p. 5, Supplementary File 4
Effect measures	12	Specify for each outcome the effect measure(s) (e.g. risk ratio, mean difference) used in the synthesis or presentation of results.	p. 7
Synthesis methods	13a	Describe the processes used to decide which studies were eligible for each synthesis (e.g. tabulating the study intervention characteristics and comparing against the planned groups for each synthesis (item #5)).	p. 6-7
	13b	Describe any methods required to prepare the data for presentation or synthesis, such as handling of missing summary statistics, or data conversions.	p. 6-7
	13c	Describe any methods used to tabulate or visually display results of individual studies and syntheses.	p. 6-7
	13d	Describe any methods used to synthesize results and provide a rationale for the choice(s). If meta-analysis was performed, describe	p. 6-7

Section and Topic	Item #	Checklist item	Location where item is reported
		the model(s), method(s) to identify the presence and extent of statistical heterogeneity, and software package(s) used.	
	13e	Describe any methods used to explore possible causes of heterogeneity among study results (e.g. subgroup analysis, meta-regression).	p. 7
	13f	Describe any sensitivity analyses conducted to assess robustness of the synthesized results.	p. 7
Reporting bias assessment	14	Describe any methods used to assess risk of bias due to missing results in a synthesis (arising from reporting biases).	NA
Certainty assessment	15	Describe any methods used to assess certainty (or confidence) in the body of evidence for an outcome.	p. 5, Supplementary File 5
**RESULTS**			
Study selection	16a	Describe the results of the search and selection process, from the number of records identified in the search to the number of studies included in the review, ideally using a flow diagram.	p. 7 & Fig. 1
	16b	Cite studies that might appear to meet the inclusion criteria, but which were excluded, and explain why they were excluded.	p. 7, Fig. 1, & Supplementary File 6
Study characteristics	17	Cite each included study and present its characteristics.	p. 7-8, Table 1, Fig. 2, Supplementary Files 7 and 8
Risk of bias in studies	18	Present assessments of risk of bias for each included study.	p. 8, Supplementary File 10, 11 and 14
Results of individual studies	19	For all outcomes, present, for each study: (a) summary statistics for each group (where appropriate) and (b) an effect estimate and its precision (e.g. confidence/credible interval), ideally using structured tables or plots.	Supplementary File 9
Results of syntheses	20a	For each synthesis, briefly summarise the characteristics and risk of bias among contributing studies.	p. 8-9, Table 2
	20b	Present results of all statistical syntheses conducted. If meta-analysis was done, present for each the summary estimate and its precision (e.g. confidence/credible interval) and measures of statistical heterogeneity. If comparing groups, describe the direction of the effect.	p. 8-10, Table 2, Figs 3-4
	20c	Present results of all investigations of possible causes of heterogeneity among study results.	p. 8-10, Table 2
	20d	Present results of all sensitivity analyses conducted to assess the robustness of the synthesized results.	p. 8-10, Table 2, Supplementary File 13
Reporting biases	21	Present assessments of risk of bias due to missing results (arising from reporting biases) for each synthesis assessed.	NA
Certainty of evidence	22	Present assessments of certainty (or confidence) in the body of evidence for each outcome assessed.	p. 8, Supplementary File 12
**DISCUSSION**			
Discussion	23a	Provide a general interpretation of the results in the context of other evidence.	p. 10
	23b	Discuss any limitations of the evidence included in the review.	p. 10-11

Section and Topic	Item #	Checklist item	Location where item is reported
	23c	Discuss any limitations of the review processes used.	p. 11-13
	23d	Discuss implications of the results for practice, policy, and future research.	p. 11-13
**OTHER INFORMATION**			
Registration and protocol	24a	Provide registration information for the review, including register name and registration number, or state that the review was not registered.	p. 4
	24b	Indicate where the review protocol can be accessed, or state that a protocol was not prepared.	p. 4
	24c	Describe and explain any amendments to information provided at registration or in the protocol.	NA
Support	25	Describe sources of financial or non-financial support for the review, and the role of the funders or sponsors in the review.	p. 14
Competing interests	26	Declare any competing interests of review authors.	p. 14
Availability of data, code and other materials	27	Report which of the following are publicly available and where they can be found: template data collection forms; data extracted from included studies; data used for all analyses; analytic code; any other materials used in the review.	p. 14

References

Page MJ, McKenzie JE, Bossuyt PM, Boutron I, Hoffmann TC, Mulrow CD, *et al.* The PRISMA 2020 statement: an updated guideline for reporting systematic reviews. BMJ. 2021;372:n71.

Supplementary File 2.

Search results for PubMed.

Search	Query	Results
#1	(((((((mental health[Title/Abstract]) OR (mental disorder[Title/Abstract])) OR (psychologic*[Title/Abstract])) OR (depression[Title/Abstract])) OR (anxiety[Title/Abstract])) OR (schizophrenia[Title/Abstract])) OR (suicide*[Title/Abstract])) OR (self-harm[Title/Abstract])	1,300,847
#2	((((temperature[Title/Abstract]) OR (heatwave[Title/Abstract])) OR (meteorologic*[Title/Abstract])) OR (climat*[Title/Abstract])) OR (warm season[Title/Abstract])	995,787
#3	(((child*) OR (adolescen*)) OR (youth))	4,864,061
#4	(((#1 AND #2 AND #3)) NOT (school climate[Title/Abstract])) NOT (qualitative[Title/Abstract])	**2,112**

Search results for Web of Science.

Search	Query	Results
#1	mental health (Title) or mental disorder (Title) or psychologic* (Title) or depression (Title) or anxiety (Title) or schizophrenia (Title) or suicide* (Title) or self-harm (Title) or mental health (Abstract) or mental disorder (Abstract) or psychologic* (Abstract) or depression (Abstract) or anxiety (Abstract) or schizophrenia (Abstract) or suicide* (Abstract) or self-harm (Abstract)	1,764,432
#2	temperature (Title) or heatwave (Title) or meteorologic* (Title) or climat* (Title) or warm season (Title) or temperature (Abstract) or heatwave (Abstract) or meteorologic* (Abstract) or climat* (Abstract) or warm season (Abstract)	5,220,012
#3	ALL=(child*) OR ALL=(adolescen*) OR ALL=(youth)	4,756,879
#4	(((((#3 AND #2 AND #1)) NOT TI=(school climate)) NOT AB=(school climate)) NOT TI=(qualitative)) NOT AB=(qualitative)	**2,282**

Search results for CINAHL Plus.

Search	Query	Results
#1	TI mental health OR TI mental disorder OR TI psychologic* OR TI depression OR TI anxiety OR TI schizophrenia OR TI suicide* OR TI self-harm	50,670
#2	AB mental health OR AB mental disorder OR AB psychologic* OR AB depression OR AB anxiety OR AB schizophrenia OR AB suicide* OR AB self-harm	96,330
#3	AB temperature OR AB heatwave OR AB meteorologic* OR AB warm season	6,116
#4	TI temperature OR TI heatwave OR TI meteorologic* OR TI warm season	1,445
#5	TI child* OR TI adolescen* OR TI youth	110,073
#6	AB child* OR AB adolescen* OR AB youth	136,724
#7	(((S1 OR S2) AND (S3 OR S4)) AND (S5 OR S6)) NOT TI school climate NOT AB school climate NOT TI qualitative NOT AB qualitative	**141**

Search results for PsycInfo.

Search	Query	Results
S1	abstract(mental health) OR abstract(mental disorder) OR abstract(psychologic*) OR abstract(depression) OR abstract(anxiety) OR abstract(schizophrenia) OR abstract(suicide*) OR abstract(self-harm)	1,038,480
S2	title(mental health) OR title(mental disorder) OR title(psychologic*) OR title(depression) OR title(anxiety) OR title(schizophrenia) OR title(suicide*) OR title(self-harm)	442,911
S3	[S1] OR [S2]	1,114,303
S4	title(temperature) OR title(heatwave) OR title(meteorologic*) OR title(climat*) OR title(warm season)	16,202
S5	abstract(temperature) OR abstract(heatwave) OR abstract(meteorologic*) OR abstract(climat*) OR abstract(warm season)	51,916
S6	[S4] OR [S5]	54,552
S7	child* OR adolescen* OR youth	1,401,396
S8	[S3] AND [S6] AND [S7]	2,217
S9	[S8] NOT school climate NOT qualitative	**1,178**

Supplementary File 3. List of review articles.

Serial no,	Record
1	Aldridge, J. M. & McChesney, K. The relationships between school climate and adolescent mental health and wellbeing: A systematic literature review. *International Journal of Educational Research* **88**, 121-145 (2018).
2	Clemens, V., von Hirschhausen, E. & Fegert, J. M. Report of the intergovernmental panel on climate change: implications for the mental health policy of children and adolescents in Europe—a scoping review. *European Child & Adolescent Psychiatry* **31**, 701-713 (2022).
3	Garcia, D. M. & Sheehan, M. C. Extreme Weather-driven Disasters and Children's Health. *International Journal of Health Services* **46**, 79-105 (2015).
4	Kunda, J. J., Gosling, S. N. & Foody, G. M. The effects of extreme heat on human health in tropical Africa. *International Journal of Biometeorology* **68**, 1015-1033 (2024).
5	Li, M., Gu, S., Bi, P., Yang, J. & Liu, Q. Heat Waves and Morbidity: Current Knowledge and Further Direction-A Comprehensive Literature Review. *International Journal of Environmental Research and Public Health* **12**, 5256-5283 (2015).
6	Liu, J. *et al.* Is there an association between hot weather and poor mental health outcomes? A systematic review and meta-analysis. *Environment International* **153**, 106533 (2021).
7	Marazziti, D. *et al.* Climate change, environment pollution, COVID-19 pandemic and mental health. *Science of The Total Environment* **773**, 145182 (2021).
8	Rother, H.-A. *et al.* Impact of extreme weather events on Sub-Saharan African child and adolescent mental health: The implications of a systematic review of sparse research findings. *The Journal of Climate Change and Health* **5**, 100087 (2022).
9	Runkle, J. R. *et al.* Socioenvironmental drivers of adolescent suicide in the United States: A scoping review. *Journal of Rural Mental Health* **47**, 65-80 (2023).
10	Thompson, R. *et al.* Ambient temperature and mental health: a systematic review and meta-analysis. *The Lancet Planetary Health* **7**, e580-e589 (2023).
11	Uibel, D., Sharma, R., Piontkowski, D., Sheffield, P. E. & Clougherty, J. E. Association of ambient extreme heat with pediatric morbidity: a scoping review. *International Journal of Biometeorology* **66**, 1683-1698 (2022).
12	Vergunst, F. & Berry, H. L. Climate Change and Children's Mental Health: A Developmental Perspective. *Clinical Psychological Science* **10**, 767-785 (2022).
13	Walinski, A. *et al.* The effects of climate change on mental health. *Deutsches Ärzteblatt International* **120**, 117 (2023).
14	Xu, Z. *et al.* Impact of ambient temperature on children's health: A systematic review. *Environmental Research* **117**, 120-131 (2012).

Supplementary File 4. Office of Health Assessment and Translation (OHAT) risk of bias rating tool

1. **Confounding bias: Did the study design or analysis account for important confounding and modifying variables?**

Definitely Low risk of bias:

There is direct evidence that appropriate adjustments or explicit considerations were made for primary covariates and confounders in the final analyses through the use of statistical models to reduce research-specific bias including standardization, matching, adjustment in multivariate model, stratification, propensity scoring, or other methods that were appropriately justified. Acceptable consideration of appropriate adjustment factors includes cases when the factor is not included in the final adjustment model because the author conducted analyses that indicated it did not need to be included,
AND there is direct evidence that primary covariates and confounders were assessed using valid and reliable measurements,
AND there is direct evidence that other exposures anticipated to bias results were not present or were appropriately measured and adjusted for. In occupational studies or studies of contaminated sites, other chemical exposures known to be associated with those settings were appropriately considered.

Probably Low risk of bias:

There is indirect evidence that appropriate adjustments were made,
OR it is deemed that not considering or only considering a partial list of covariates or confounders in the final analyses would not appreciably bias results.
AND there is evidence (direct or indirect) that primary covariates and confounders were assessed using valid and reliable measurements, OR it is deemed that the measures used would not appreciably bias results (i.e., the authors justified the validity of the measures from previously published research),
AND there is evidence (direct or indirect) that other co-exposures anticipated to bias results were not present or were appropriately adjusted for, OR it is deemed that co-exposures present would not appreciably bias results.
Note: As discussed above, this includes insufficient information provided on co-exposures in general population studies.

Probably High risk of bias:

There is indirect evidence that the distribution of primary covariates and known confounders differed between the groups and was not appropriately adjusted for in the final analyses,
OR there is insufficient information provided about the distribution of known confounders (record "NR" as basis for answer),
OR there is indirect evidence that primary covariates and confounders were assessed using measurements of unknown validity,
OR there is insufficient information provided about the measurement techniques used to assess

primary covariates and confounders (record "NR" as basis for answer),
OR there is indirect evidence that there was an unbalanced provision of additional co-exposures across the primary study groups, which were not appropriately adjusted for,
OR there is insufficient information provided about co-exposures in occupational studies or studies of contaminated sites where high exposures to other chemical exposures would have been reasonably anticipated (record "NR" as basis for answer).

Definitely High risk of bias:

There is direct evidence that the distribution of primary covariates and known confounders differed between the groups, confounding was demonstrated, and was not appropriately adjusted for in the final analyses,
OR there is direct evidence that primary covariates and confounders were assessed using non valid measurements,
OR there is direct evidence that there was an unbalanced provision of additional co-exposures across the primary study groups, which were not appropriately adjusted for.

2. Detection Bias: Can we be confident in the exposure characterization?

Definitely Low risk of bias:

There is direct evidence that exposure was consistently assessed (i.e., under the same method and time-frame) using well-established methods that directly measure exposure (e.g., measurement of the chemical in air or measurement of the chemical in blood, plasma, urine, etc.),
OR exposure was assessed using less-established methods that directly measure exposure and are validated against well-established methods.

Probably Low risk of bias:

There is indirect evidence that the exposure was consistently assessed using well-established methods that directly measure exposure,
OR exposure was assessed using indirect measures (e.g., questionnaire or occupational exposure assessment by a certified industrial hygienist) that have been validated or empirically shown to be consistent with methods that directly measure exposure (i.e., inter-methods validation: one method vs. another).

Probably High risk of bias:

There is indirect evidence that the exposure was assessed using poorly validated methods that directly measure exposure,
OR there is direct evidence that the exposure was assessed using indirect measures that have not been validated or empirically shown to be consistent with methods that directly measure exposure (e.g., a job-exposure matrix or self-report without validation) (record "NR" as basis for answer),
OR there is insufficient information provided about the exposure assessment, including validity and reliability, but no evidence for concern about the method used (record "NR" as basis for

answer).

Definitely High risk of bias:

There is direct evidence that the exposure was assessed using methods with poor validity,
OR evidence of exposure misclassification (e.g., differential recall of self-reported exposure).

3. Detection Bias: Can we be confident in the outcome assessment?

Definitely Low risk of bias:

There is direct evidence that the outcome was assessed using well-established methods (the gold standard),
AND there is direct evidence that the outcome assessors (including study subjects, if outcomes were self-reported) were adequately blinded to the exposure level, and it is unlikely that they could have broken the blinding prior to reporting outcomes

Probably Low risk of bias:

There is indirect evidence that the outcome was assessed using acceptable methods,
OR it is deemed that the outcome assessment methods used would not appreciably bias results,
AND there is indirect evidence that the outcome assessors were adequately blinded to the exposure level, and it is unlikely that they could have broken the blinding prior to reporting outcomes,
OR it is deemed that lack of adequate blinding of outcome assessors would not appreciably bias results (including that subjects self-reporting outcomes were likely not aware of reported links between the exposure and outcome lack of blinding is unlikely to bias a particular outcome).

Probably High risk of bias:

There is indirect evidence that the outcome assessment method is an insensitive instrument,
OR there is indirect evidence that it was possible for outcome assessors to infer the exposure level prior to reporting outcomes (including that subjects self-reporting outcomes were likely aware of reported links between the exposure and outcome),
OR there is insufficient information provided about blinding of outcome assessors (record "NR" as basis for answer).

Definitely High risk of bias:

There is direct evidence that the outcome assessment method is an insensitive instrument,
OR there is direct evidence that outcome assessors were aware of the exposure level prior to reporting outcomes (including that subjects self-reporting outcomes were aware of reported links between the exposure and outcome).

4. **Selective reporting of results: Were all measured outcomes reported?**

Definitely Low risk of bias:

There is direct evidence that all of the study's measured outcomes (primary and secondary) outlined in the protocol, methods, abstract, and/or introduction (that are relevant for the evaluation) have been reported. This would include outcomes reported with sufficient detail to be included in meta-analysis or fully tabulated during data extraction and analyses had been planned in advance.

Probably Low risk of bias:

There is indirect evidence that all of the study's measured outcomes (primary and secondary) outlined in the protocol, methods, abstract, and/or introduction (that are relevant for the evaluation) have been reported,
OR analyses that had not been planned in advance (i.e., retrospective unplanned subgroup analyses) are clearly indicated as such and it is deemed that the unplanned analyses were appropriate and selective reporting would not appreciably bias results (e.g., appropriate analyses of an unexpected effect). This would include outcomes reported with insufficient detail such as only reporting that results were statistically significant (or not).

Probably High risk of bias:

There is indirect evidence that all of the study's measured outcomes (primary and secondary) outlined in the protocol, methods, abstract, and/or introduction (that are relevant for the evaluation) have been reported,
OR and there is indirect evidence that unplanned analyses were included that may appreciably bias results,
OR there is insufficient information provided about selective outcome reporting (record "NR" as basis for answer).

Definitely High risk of bias:

There is direct evidence that all of the study's measured outcomes (primary and secondary) outlined in the protocol, methods, abstract, and/or introduction (that are relevant for the evaluation) have not been reported. In addition to not reporting outcomes, this would include reporting outcomes based on composite score without individual outcome components or outcomes reported using measurements, analysis methods or subsets of the data (e.g., subscales) that were not pre-specified or reporting outcomes not pre-specified, or that unplanned analyses were included that would appreciably bias results.

5. **Other Bias - Were there no other potential threats to internal validity (e.g., statistical methods were appropriate and researchers adhered to the study protocol)?**

Definitely Low risk of bias:

The study appears to be free of other sources of bias.

PROBABLY Low risk of bias:

There is insufficient information to permit a judgment of definitely low risk of bias, but there is indirect evidence that suggests the study was free of other threats to validity.

PROBABLY High risk of bias:

There is insufficient information to permit a judgment of definitely high risk of bias, but there is indirect evidence that suggests the study was not free of other threats to validity, as described by the criteria for a judgment of high risk of bias.

Definitely High risk of bias:
There is at least one important risk of bias. For example, the study:
- Had a potential source of bias related to the specific study design used; or
- Stopped early due to some data-dependent process (including a formal-stopping rule); or
- The conduct of the study is affected by interim results (e.g. recruiting additional participants from a subgroup showing greater or lesser effect); or
- Has been claimed to have been fraudulent; or
- Had some other problem

References

Johnson PI, Koustas E, Vesterinen HM, Sutton P, Atchley DS, Kim AN, *et al.* Application of the Navigation Guide systematic review methodology to the evidence for developmental and reproductive toxicity of triclosan. Environment International. 2016;92-93:716-28.

National Toxicology Program US Department of Health and Human Services National Institutes of Health. OHAT Risk of Bias Rating Tool for Human and Animal Studies. 2015.

Supplementary File 5. Grading of Recommendations Assessment, Development and Evaluation (GRADE) guideline.

	Domain	Definition	Typical example	Graading
Downgrading dimension	Risk of bias	Downgrade if limitations biasing the exposure effect exist, which can be taken reference from quality assessments for individual studies.	Subjectively measured outcomes or exposures	-1 if serious;<br>-2 if very serious
	Indirectness	Downgrade if the evidence associated with population, exposure, comparator and outcome is not comparable to the pre-defined research question.	- Participants differ from the population of interest<br>- Using a proxy for outcome or exposure measurements	-1 if serious;<br>-2 if very serious
	Inconsistency	Downgrade if differing effect estimates exist without observable reasons.	- The effect estimates are particularly higher or lower among a specific subgroup<br>- The effect estimates are particularly higher or lower at a specific time-point	-1 if serious;<br>-2 if very serious
	Imprecision	Downgrade if wide confidence intervals are detected.	-	-1 if serious;<br>-2 if very serious
	Publication bias	Downgrade if studies are found to report findings selectively.	-	-1 if likely;<br>-2 if very likely
Upgrading dimension	Large magnitude of effect	Upgrade if significant increment or decrement in effect estimates were observed.	- Increment or decrement at or greater than two-fold in effect estimates<br>- Increment or decrement at or greater than five-fold in effect estimates	+1 if large;<br>+2 if very large
	Dose response	Upgrade if a dose-response relationship	-	+1 for evidence of a gradient

		between the exposure and outcome is observed.		
	Confounding minimizes effect	Upgrade if potential residual confounding would additionally support inferences of the effect induced by exposure.	-	+1 for evidence of effect

References

Guyatt GH, Oxman AD, Kunz R, Vist GE, Falck-Ytter Y, Schünemann HJ. What is "quality of evidence" and why is it important to clinicians? *BMJ*. 2008;336(7651):995.

Supplementary File 6. List of excluded articles after full-text review.

Serial no.	Record	Exclusion reason	Identification method
1	Xu, R. *et al.* Socioeconomic level and associations between heat exposure and all-cause and cause-specific hospitalization in 1,814 Brazilian cities: A nationwide case-crossover study. *PLOS Medicine* **17**, e1003369 (2020).	No analysis for young people	Databases
2	Slater, P. Climate change: the psychological impact of climate anxiety and trauma: understanding from the psychotherapeutic encounter. *Journal of Child Psychotherapy* **49**, 490-508 (2023).	Qualitative research	Databases
3	Nori-Sarma, A. *et al.* Association Between Ambient Heat and Risk of Emergency Department Visits for Mental Health Among US Adults, 2010 to 2019. *JAMA Psychiatry* **79**, 341-349.2021.4369 (2022).	Adults	Databases
4	Wang, X., Lavigne, E., Ouellette-kuntz, H. & Chen, B. E. Acute impacts of extreme temperature exposure on emergency room admissions related to mental and behavior disorders in Toronto, Canada. Journal of Affective Disorders **155**, 154-161 (2014).	No analysis for young people	Databases
5	Wu, Y.-W., Chen, C.-K. & Wang, L.-J. Is suicide mortality associated with meteorological and socio-economic factors? An ecological study in a city in Taiwan with a high suicide rate. Psychiatria Danubina 26, 0-158 (2014).	No analysis for young people	Databases
6	Vida, S., Durocher, M., Ouarda, T. B. & Gosselin, P. Relationship between ambient temperature and humidity and visits to mental health emergency departments in Québec. Psychiatric Services 63, 1150-1153 (2012).	No analysis for young people	Databases
7	Huibers, M. J. H., de Graaf, L. E., Peeters, F. P. M. L. & Arntz, A. Does the weather make us sad? Meteorological determinants of mood and depression in the general population. *Psychiatry Research* **180**, 143-146 (2010).	No analysis for young people	Databases
8	Walinski, A. *et al.* The effects of climate change on mental health. *Deutsches Ärzteblatt International* **120**, 117 (2023).	Review	Databases
9	Yarza, S. *et al.* Suicide behavior and meteorological characteristics in hot and arid climate. *Environmental Research* **184**, 109314 (2020).	No analysis for young people	Databases
10	Squires, E., Whiting, L. & Petty, J. Effects of climate change on the health of children and young people. *Nursing Standard* (2024).	Review	Databases
11	Gibson, K. E., Barnett, J., Haslam, N. & Kaplan, I. The mental health impacts of climate change: Findings from a Pacific Island atoll nation. *Journal of Anxiety Disorders* **73**, 102237 (2020).	Qualitative research	Databases

12	Dang, T. N. *et al.* Main and added effects of heatwaves on hospitalizations for mental and behavioral disorders in a tropical megacity of Vietnam. *Environmental Science and Pollution Research* **29**, 59094-59103 (2022).	No analysis for young people	Databases
13	Park, J., Kim, A., Bell, M. L., Kim, H. & Lee, W. Heat and hospital admission via the emergency department for people with intellectual disability, autism, and mental disorders in South Korea: a nationwide, time-stratified, case-crossover study. *The Lancet Psychiatry* **11**, 359-367 (2024).	No analysis for young people	Databases
14	Majeed, H. & Lee, J. The impact of climate change on youth depression and mental health. *The Lancet Planetary Health* **1**, e94-e95 (2017).	Comment	Databases
15	Yu, L. *et al.* The role of meteorological factors in suicide mortality in Wuhu, a humid city along the Yangtze River in Eastern China. *Environmental Science and Pollution Research* **30**, 9558-9575 (2023).	No analysis for young people	Databases
16	Williams, M. N., Hill, S. R. & Spicer, J. Do hotter temperatures increase the incidence of self-harm hospitalisations? *Psychology, Health & Medicine* **21**, 226-235 (2016).	No analysis for young people	Databases
17	Basagaña, X. *et al.* Heat Waves and Cause-specific Mortality at all Ages. *Epidemiology* **22** (2011).	No analysis for young people	Databases
18	Sung, T.-I., Chen, M.-J., Lin, C.-Y., Lung, S.-C. & Su, H.-J. Relationship between mean daily ambient temperature range and hospital admissions for schizophrenia: Results from a national cohort of psychiatric inpatients. *Science of The Total Environment* **410-411**, 41-46 (2011).	No analysis for young people	Databases
19	Corvetto, J. F. *et al.* Private vs. public emergency visits for mental health due to heat: An indirect socioeconomic assessment of heat vulnerability and healthcare access, in Curitiba, Brazil. *Science of The Total Environment* **934**, 173312 (2024).	No analysis for young people	Databases
20	Hauser, L.-L., Höner, O. & Wachsmuth, S. Links between environmental features and developmental outcomes of elite youth athletes: A cross-sectional study within the German talent pathway. *Psychology of Sport and Exercise* **71**, 102569 (2024).	Irrelevant outcome	Databases
21	Burke, S. E. L., Sanson, A. V. & Van Hoorn, J. The Psychological Effects of Climate Change on Children. *Current Psychiatry Reports* **20**, 35 (2018).	Review	Databases
22	Barkin, J. L. *et al.* Effects of extreme weather events on child mood and behavior. *Developmental Medicine & Child Neurology* **63**, 785-790 (2021).	Review	Databases
23	Sherbakov, T., Malig, B., Guirguis, K., Gershunov, A. & Basu, R. Ambient temperature and added heat wave	No analysis for young people	Databases

	effects on hospitalizations in California from 1999 to 2009. *Environmental Research* **160**, 83-90 (2018).		
24	Zhou, Y.-M. *et al.* Association between short-term ambient air pollution exposure and depression outpatient visits in cold seasons: a time-series analysis in northwestern China. *Journal of Toxicology and Environmental Health*, Part A **84** (2021).	No analysis for young people	Databases
25	Provenzi, L. *et al.* Climate Change Challenge Faced by Italian Children: A Nationwide Study. *Healthcare* **12** (2024).	Irrelevant exposure	Databases
26	Alho, A. M., Oliveira, A. P., Viegas, S. & Nogueira, P. Effect of heatwaves on daily hospital admissions in Portugal, 2000-18: an observational study. *The Lancet Planetary Health* **8**, e318-e326 (2024).	No analysis for young people	Databases
27	Middleton, J. *et al.* Temperature and place associations with Inuit mental health in the context of climate change. *Environmental Research* **198**, 111166 (2021).	No analysis for young people	Databases
28	Xu, Z., FitzGerald, G., Guo, Y., Jalaludin, B. & Tong, S. Assessing heatwave impacts on cause-specific emergency department visits in urban and rural communities of Queensland, Australia. *Environmental Research* **168**, 414-419 (2019).	No analysis for young people	Databases
29	Kunda, J. J., Gosling, S. N. & Foody, G. M. The effects of extreme heat on human health in tropical Africa. *International Journal of Biometeorology* **68**, 1015-1033 (2024).	Review	Databases
30	Kordić, M. *et al.* The meteorological factors associated with suicide. *Collegium antropologicum* **34**, 151-155 (2010).	No analysis for young people	Databases
31	Jee, H.-J. *et al.* Solar radiation increases suicide rate after adjusting for other climate factors in South Korea. *Acta Psychiatrica Scandinavica* **135**, 219-227 (2017).	No analysis for young people	Databases
32	Gupta, S. & Murray, R. M. The Relationship of Environmental Temperature to the Incidence and Outcome of Schizophrenia. *British Journal of Psychiatry* **160**, 788-792 (1992).	No analysis for young people	Databases
33	Garcia, D. M. & Sheehan, M. C. Extreme Weather-driven Disasters and Children's Health. *International Journal of Health Services* **46**, 79-105 (2015).	Review	Citation search
34	Mayner, L., Arbon, P. & Usher, K. Emergency department patient presentations during the 2009 heatwaves in Adelaide. Collegian 17, 175-182 (2010).	No analysis for young people	Citation search
35	Franklin, R. C. *et al.* Heatwaves and mortality in Queensland 2010–2019: implications for a homogenous state-wide approach. International *Journal of Biometeorology* **67**, 503-515 (2023).	No analysis for young people	Citation search
36	Hertzog, L. *et al.* Suicide deaths associated with climate change-induced heat anomalies in Australia: a time	No analysis for young people	Citation search

	series regression analysis. *BMJ Mental Health* **27**, e301131(2024).		
37	Likhvar, V., Honda, Y. & Ono, M. Relation between temperature and suicide mortality in Japan in the presence of other confounding factors using time-series analysis with a semiparametric approach. *Environmental Health and Preventive Medicine* **16**, 36-43 (2011).	No analysis for young people	Citation search
38	Kayipmaz, S., San, I., Usul, E. & Korkut, S. The effect of meteorological variables on suicide. *International Journal of Biometeorology* **64**, 1593-1598 (2020).	No analysis for young people	Citation search
39	Kalkstein, A. J., Belorid, M., Dixon, P. G., Kim, K. R. & Bremer, K. A. Seasonal Variations in Temperature–Suicide Associations across South Korea. *Weather, Climate, and Society* **11**, 731-739 (2019).	No analysis for young people	Citation search
40	Díaz-Castro, S. C. & Zenteno-Savín, T. Ambient temperature increase and its possible association with suicide in the Population of Baja California Sur (BCS) 1985-2008. *Salud mental* **36**, 421-427 (2013).	No analysis for young people	Citation search
41	Fernández-Niño, J. A., Flórez-García, V. A., Astudillo-García, C. I. & Rodríguez-Villamizar, L. A. Weather and suicide: a decade analysis in the five largest capital cities of Colombia. *International journal of environmental research and public health* **15**, 1313 (2018).	No analysis for young people	Google
42	Vercammen A, Wray B, Crider YS, *et al.* Psychological impacts of climate change on US youth. *Proceedings of the National Academy of Sciences of the United States of America* **22**;122(16) (2025).	Irrelevant exposure	Citation search
43	Seastedt, H., Schuetz, J., Perkins, A., Gamble, M., & Sinkkonen A. Impact of urban biodiversity and climate change on children's health and well being. *Pediatric Research* 98, 452–457 (2025).	Review	Citation search
44	Torales J, Laterza M, Persaud A, *et al.* Association between ambient temperature and emergency psychiatric consultations: A case-crossover study in a South American emergency setting (2021–2023). *International Journal of Social Psychiatry* **71**(6):1181-1192 (2025).	No analysis for young people	Citation search
45	Casas, L., Cox, B., Nemery, B. *et al.* High temperatures trigger suicide mortality in Brussels, Belgium: A case-crossover study (2002–2011). *Environmental Research* 207 (2022).	No analysis for young people	Citation search
46	Park, J., Oh, J., Lee, W. *et al.* Association of ambient temperatures with suicide attempts and violence with the future projections under climate change scenarios: a nationwide time-stratified case-crossover study in South Korea. BMC Public Health 25, 457 (2025).	No analysis for young people	Citation search

Supplementary File 7. List of included studies for systematic review.

Serial no,	Record
1	Basu, R., Gavin, L., Pearson, D., Ebisu, K. & Malig, B. Examining the Association Between Apparent Temperature and Mental Health-Related Emergency Room Visits in California. *American Journal of Epidemiology* **187**, 726-735 (2018).
2	Bernstein Aaron, S. *et al.* Warm Season and Emergency Department Visits to U.S. Children's Hospitals. *Environmental Health Perspectives* **130**, 017001 (2022).
3	Chan, E. Y. Y. *et al.* Association between Ambient Temperatures and Mental Disorder Hospitalizations in a Subtropical City: A Time-Series Study of Hong Kong Special Administrative Region. *International Journal of Environmental Research and Public Health* **15** (2018).
4	Cohen, G. *et al.* Daily temperature variability and mental health-related hospital visits in New York State. *Environmental Research* **257**, 119238 (2024).
5	da Silva, I., de Almeida, D. S., Hashimoto, E. M. & Martins, L. D. Risk assessment of temperature and air pollutants on hospitalizations for mental and behavioral disorders in Curitiba, Brazil. *Environmental Health* **19**, 79 (2020).
6	He, W.-Q. *et al.* Extreme Heat Stress and Unplanned Hospital Admissions. *Pediatrics* **155**, e2024068183 (2024).
7	Hu, J. *et al.* Associations of exposure to heatwaves with depression and anxiety among adolescents: A cross-sectional study of the Chinese adolescent health survey. Journal of Affective Disorders **387**, 119499 (2025).
8	Isaksen, T. B. *et al.* Increased mortality associated with extreme-heat exposure in King County, Washington, 1980–2010. *International Journal of Biometeorology* **60**, 85-98 (2016).
9	Kim, Y. *et al.* Suicide and Ambient Temperature in East Asian Countries: A Time-Stratified Case-Crossover Analysis. *Environmental Health Perspectives* **124**, 75-80 (2016).
10	Komulainen, K. *et al.* Climatic exposures in childhood and the risk of schizophrenia from childhood to early adulthood. *Schizophrenia Research* **248**, 233-239 (2022).
11	Mullins, J. T. & White, C. Temperature and mental health: Evidence from the spectrum of mental health outcomes. *Journal of Health Economics* **68**, 102240 (2019).
12	Florido Ngu, F., Kelman, I., Chambers, J. & Ayeb-Karlsson, S. Correlating heatwaves and relative humidity with suicide (fatal intentional self-harm). *Scientific Reports* **11**, 22175 (2021).
13	Ndovu, A. *et al.* Spatial Variation in the Association between Extreme Heat Events and Warm Season Pediatric Acute Care Utilization: A Small-Area Assessment of Multiple Health Conditions and Environmental Justice Implications in California (2005–2019). *Environmental Health Perspectives* **133**, 017010 (2025).
14	Nitschke, M., Tucker, G. R. & Bi, P. Morbidity and mortality during heatwaves in metropolitan Adelaide. *Medical Journal of Australia* **187**, 662-665 (2007).
15	Niu, L. *et al.* Temperature and mental health–related emergency department and hospital encounters among children, adolescents and young adults. *Epidemiology and Psychiatric Sciences* **32**, e22 (2023).
16	Niu, Y. *et al.* Short-term effect of apparent temperature on daily emergency visits for mental and behavioral disorders in Beijing, China: A time-series study. *Science of The Total Environment* **733**, 139040 (2020).
17	Parks, R. M. *et al.* Anomalously warm temperatures are associated with increased injury deaths. *Nature Medicine* **26**, 65-70 (2020).

18	Rahman, M. M. *et al.* Ambient temperature and air pollution associations with suicide and homicide mortality in California: A statewide case-crossover study. *Science of The Total Environment* **874**, 162462 (2023).
19	Runkle, J. D. *et al.* Assessing the impact of heatwaves on emergency visits for major depression and suicidal ideation in youth with attention-deficit/hyperactivity disorder. *PLOS Mental Health* **2**, e0000444 (2025).
20	Stowell, J. D. *et al.* Warm-season temperatures and emergency department visits among children with health insurance. *Environmental Research: Health* **1**, 015002, (2023).
21	Trang, P. M., Rocklöv, J., Giang, K. B., Kullgren, G. & Nilsson, M. Heatwaves and Hospital Admissions for Mental Disorders in Northern Vietnam. *PLOS ONE* **11**, e0155609 (2016).
22	Villeneuve, P. J. *et al.* Daily changes in ambient air pollution concentrations and temperature and suicide mortality in Canada: Findings from a national time-stratified case-crossover study. *Environmental Research* **223**, 115477 (2023).
23	Wang, S. *et al.* Effect of increasing temperature on daily hospital admissions for schizophrenia in Hefei, China: a time-series analysis. *Public Health* **159**, 70-77 (2018).
24	Xu, Y., Wheeler, S. A. & Zuo, A. Will boys' mental health fare worse under a hotter climate in Australia? *Population and Environment* **40**, 158-181 (2018).
25	Zhang, S. *et al.* The effect of temperature on cause-specific mental disorders in three subtropical cities: A case-crossover study in China. *Environment International* **143**, 105938 (2020).
26	Zhong Z, Xu J, Liu Z, *et al.* The impact of different types of extreme temperature events on mental disorders: A case-crossover study in Anhui Province, China. *Environmental Research* 2025; **277**: 121526.
27	Zhou, Y. *et al.* The role of extreme high humidex in depression in chongqing, China: A time series-analysis. *Environmental Research* **222**, 115400 (2023).
28	Zhou, Q. *et al.* Immediate and delayed effects of environmental temperature on schizophrenia admissions in Liuzhou, China, 2013–2020: a time series analysis. *International Journal of Biometeorology* **68**, 843-854 (2024).

Supplementary File 8. Systematic review

Study profile

A total of 28 studies published between 2007 and 2025 were included for systematic review (Table 1). These included 13 studies of time-series design (46%)[1-13], ten of case-crossover design (36%)[14-23], three of cohort design (11%)[24-26], one of matched-case design (4%)[27], and one of ecological design (4%)[28]. Full study profile for individual studies were presented in Supplementary Tables 9-36. Stratifying by geographical settings, 11 studies were conducted in the US[1,2,5,7,9,15,16,19,21,23,27], seven in Mainland China[6,8,10,11,18,22,26], three in Australia[13,20,25], and one each in Brazil[4], Canada[17], Finland[24], Hong Kong[3], Vietnam[12], Japan, South Korea and Taiwan[14]. One multi-country study was conducted across sixty countries, excluding US, China, India and Russia[28]. All of the included studies comprised both boys and girls aged 24 years or younger. In terms of study duration, nine of 13 time-series studies involved cases over less than or equal to a 10-year period (mean duration=6.7, range: 3-10 years)[1-4,6,8,10-12], while four studies employed data of more than 10 years (mean=30.4, range: 12-57 years)[5,7,9,13]. For the case-crossover studies, five studies used data less than a 10-year period (mean=4.6, range: 3-7 years)[15,16,18,22,23], whereas five studies employed data of more than 10 years (mean=20.7, range: 14-39 years)[14,17,19-21]. The two cohort studies included one of longitudinal design that followed up individuals from age 10 until early adulthood[24] and another two of cross-sectional design[25,26]. The study of retrospective matched-case design analyzed data over 14 years[27], whereas the one of ecological design 38 years[28].

Outcome assessments

In terms of outcome measures, the 13 time-series studies used 17 mental health-related outcomes. Eight studies examined hospital visits or hospitalizations for mental health disorders[1-4,6,9,12,13], two studies solely considered schizophrenia[10,11], and one considered depression[8]. One study considered neurotic disorders, and self-inflicted injury or suicide[1], and another considered suicidality and depression[2]. Three studies considered mortality outcomes, with two focusing on suicide deaths[7,9] and another on mortality associated with mental disorders[5]. The ten case-crossover studies considered 33 various mental health-related outcomes. Five studies used hospital visits or hospitalizations for mental health disorders as outcome[15,20-23], with three studies additionally considered suicidality and depression[20,21,23]. Of the three studies that explored both mental health disorders and, suicidality and depression, one analyzed data independently for emergency department visits and hospital admission[20]. Four studies employed hospital visits or hospitalizations for anxiety[15,18,19,22], three focused on depression[15,18,22], and three focused on

schizophrenia or other psychotic disorders[18,19,22]. One study additionally considered mood disorders and adjustments disorders[19], whereas another study focused on organic mental disorders and affective disorders[18]. Other mental health-related outcomes including externalizing disorders, reaction disorders, psychosis, bipolar disorder and substance abuse, and suicide and self-inflicted injury were also considered in one study[15]. There were three studies that considered suicidal mortality as an outcome[14,16,17]. For the three cohort studies, one used hospital visits or hospitalizations for schizophrenia[24], one measured mental health based on the Strengths and Difficulties Questionnaire[25] and another one measured depression using the 9-item Patient Health Questionnaire (PHQ-9) and anxiety using the 7-item Generalized Anxiety Disorder (GAD-7)[26]. The only retrospective matched-case study used hospital visits or hospitalizations for depression and suicidal behaviour[27]. The single study of ecological design explored suicide defined as fatal intentional self-harm[28].

The outcome measures of the time-series, case-crossover, retrospective matched-case and cohort studies included in the analysis were mostly defined as per the ICD-9 (n=3)[1,3,15], ICD-10 (n=12)[4,6,8,10-12,16-18,22-24], ICD-9, clinical modification (CM) (n=1)[19], ICD-10-Australian modification (AM) (n=1)[20], ICD-10-CM (n=1)[2]. There were five studies that considered both ICD-9 and ICD-10[5,7,13,21,27], one study that considered ICD-9-CM and ICD-10[9], while another study that considered ICD-8, ICD-9 and ICD-10[14]. One study additionally employed ICD-7[9]. The study with ecological design used data derived from the WHO's mortality database, which employed multiple systems from ICD-7 to ICD-10[28].

Exposure assessments

Exposure measures varied across the included studies. Within the 13 time-series studies, eight studies employed temperature data derived from meteorological monitoring stations[1,3,4,6,8,10-12]. Of these eight studies, seven studies aggregated the data to calculate city-level mean temperature, whereas one study calculated climate-zone level temperature[1]. Four of the 13 time-series studies used gridded data at varied spatial resolutions of $4\times4Km^2$ [2,9], $7\times7Km^2$ (or 1/16°)[5] or $30\times30Km^2$ [7]. By using these gridded datasets, three studies aggregated the pixels to create county-level temperature measures, whereas one study measured state-level temperature [7]. The remaining study did not specify the exposure measures in the original article[13]. Among the ten case-crossover studies, three studies employed data from meteorological monitoring stations and then aggregated the data to calculate city-level temperature[14,15,18]. Six studies employed gridded data for exposure measures, with three studies employing $4\times4Km^2$ resolution data for each residential census tract[16,23] or ZIP

code[21], and one study employing 11x14Km^2 resolution data for each ZIP code[19]. One study measured temperature using a global dataset (ERA5) developed using data from meteorological monitoring stations and satellite[20], whereas one study used the CN05.1 grid data[22]. There was one study that measured temperature using interpolated metrics available for all postal codes[17]. Three of the cohort studies used gridded data, with one employing 10×10Km^2 resolution data for each residential zip code[24], one using 1×1Km^2 resolution data for each postcode[25], and another one using ERA5[26]. The study of retrospective matched-case design employed the nClimGrid product[27]. The ecological study measured country-level temperature using ERA5[28].

There was marked heterogeneity in the exposure metrics used across the pooled studies. Of the 13 time-series studies, three studies employed continuous temperature exposure metrics. Of these, one study examined the effects of 5.6°C (10°F) increment in summertime daily mean temperature[1], one study examined the effects of 1°F increment in mean monthly temperature[9], while another considered 1.5°C increment in the deviations of monthly temperature from the local average monthly temperature over the study period[7]. Eight studies examined the effects of exposure to high temperature extremes, with two studies defining heatwaves as temperature exceeding 35°C[12,13], one study defining high temperature based on the 95th percentile of the maximum temperature distribution during summertime[2], one defining it based on the 90th percentile of the daily mean temperature[6], two considering the 75th percentile of the daily mean temperature[3,10], one considering daily mean temperature in reference to temperature at 22.4°C[4], while, another considered high temperature in reference to 75th percentile at 21.7°C[11]. Two studies employed a temperature index called *humidex* that additionally accounted for humidity. Of these, one defined high humidex as a value greater than 40[8], while the other defined heat day based on the 99th percentile of the average area-wide maximum humidex value[5]. Among the ten case-crossover studies, three studies employed a continuous temperature metrics, with one examining the effect of one standard deviation increment in each city's mean temperature divided by 2[14], one considering effect of 1°C increase in daily minimum and maximum temperature[16], and another study investigating an interquartile range (9.6°C) increase in the average daily temperature[1]. Seven studies examined the effects of high temperature exposures, with one study defining high temperature in terms of 97.5th percentile of temperature[18], and three studies defining it based on the 95th percentile of the distribution of the daily minimum temperature during warm seasons[15,21,23], one study defining it based on both maximum and minimum temperature at 90th percentile[22], one defined it based on a wide-range of cut-off points ranging between 1st and 99th percentile[19], while one created heatwave metrics using the Universal Thermal Climate Index[20]. One

of the cohort studies measured the effects for each 1°C increase in annual mean maximum temperature[25], one categorized the temperature based on quartiles[24], and another one defined heatwave using the excess heat factor[26]. The retrospective matched-case study defined heatwave using the excess heat factor[27]. The only ecological study explored heat waves defined as periods with four or more continuous days with temperature exceeding the 99th percentile of temperature data[28].

The choice of lag period defined as the time interval between temperature exposure and mental health event onset varied across the included studies. Of the 13 time-series studies, nine studies measured same-day temperature exposure as the event (lag0)[1,4-6,8,10-13], with six of these studies additionally examining other lag structures. Of these six studies, four studies used both multi-single lag-day (lag1, lag2…lagn) and a period-based (lag01, lag02…lag0n) lag structure, with restrictions of the time period to 6[10], 7[6] or 14 lag days[8,11]. One study measured temperature over periods of lag01 and lag07[4], while another one measured temperature over lag03 and lag07[28]. Of the remaining four time-series studies, one study measured temperature over a period of lag02[3], one study considered lag07[2], and two considered monthly mean temperature[7,9]. In the case-crossover studies, five studies measured same-day temperature[14,16-18,21], with three of these studies additionally exploring other lag structures. Of these three studies, one study explored 5 lag periods (lag01, lag03, lag05, lag07,lag09)[18], one used a multi-single lag-day structure over a 7–day period[16], and one used both period-based and multi-single-day structures with a limit of 2 lag days[17]. The remaining five studies measured temperature over a period of either lag03[20], lag05[15,23], lag06[19], or lag021[22]. For the three cohort studies, one measured temperature from birth to 10th birthday[24], one measured 12-month temperature prior to the survey assessment[26], and another one measured annual average temperature[25]. The retrospective matched-case study used both multi-single lag day (lag0, lag1…lag7) and multiple period-based lag (lag03, lag05, lag07) structures[27]. The ecological study measured annual mean temperature[28].

Statistical models

Of the 13 time-series studies, two studies used Poisson regression[1,13]. Eight studies employed distributed lag nonlinear model (DLNM) to account for non-linearity in the exposure-outcome association in combinations with generalized additive model (n=3)[3,4,6], Poisson-generalized linear model (n=2)[10,11], quasi-Poisson model (n=2)[2,8], or piecewise linear and Poisson regression model (n=1)[5]. One study each employed zero-inflated negative binomial regression model[12] and panel fixed effects method[9]. Another study employed the Bayesian spatio-temporal model accounting for

spatiotemporal autocorrelation[7]. Among the ten case-crossover studies, eight studies used conditional logistic regression[14-17,19,21-23], with six studies additionally using DLNM[15,16,19,21-23]. Two studies used quasi-Poisson generalized linear model in combination with DLNM[18,20]. The three cohort studies employed either Cox proportional hazards model[24], logistic regression[26] or structural equation modelling[25]. The retrospective matched-cased study employed poisson mixed-effect regression models[27]. The study with ecological design employed negative binomial regression models[28].

The 13 time-series studies employed diverse approaches to account for seasonal and/or long-term trend in their models. Seven studies adjusted for time calculated in years using a natural cubic spline with 3 degree of freedom (df) per year[10], 4 df per year[6], 7 df per year[4,8], 8 df per year[11], 16 df per year[12], or 19 df over 31 years[5]. One study used a spline function calculated in days with 2 df for each season[1], whereas one study used a natural cubic B-spline of time with nearly 1 df for every 10 years to control for long-term trend, and a natural cubic B-spline calculated in days during warm season with 4 df for each year for seasonal trend adjustment[2]. One study adjusted for day of study and day of year[3], whereas another study adjusted for county-by-month and county/state-by-year fixed effects[9]. The study that employed Bayesian spatio-temporal model took into account the month of year and overall time in months[7]. The remaining study excluded autumn and winter to account for seasonality[13]. The ten case-crossover studies adjusted for long-term trend and seasonal patterns by design. Nine studies matched each case with 3 or 4 control days on the same day of a week within the same month[15-23]. One study matched each case with 7 control days on the same day of the week over a 56-day time window, and adjusted for the month and date of event (suicide)[14]. The retrospective matched-case study included a random intercept and fixed effects for day of week, year and a binary variable to control for warm season trends[27].

Covariate adjustments

Majority of the time-series studies adjusted for day of the week and holiday (n=8)[1-4,6,8,10,11]. Some studies adjusted for climatic variables including precipitation (n=6)[3,6,8-11], relative humidity (n=4)[2-4,11], and air pollutant (n=3)[4,6,8]. Seven out of ten case-crossover studies adjusted for relative humidity[14-18,22,23]. Some studies adjusted for holidays including weekends and air pollutant (n=3)[17,18,22].

Supplementary File 9. Study characteristics of individual studies

Study characteristics of Basu *et al.,* 2018

Characteristics	Details
Study design	Time-series
Study year	2005-2013
Geographical setting	California, US
Sex	Both
Age (year)	6-18
Sample size	219,942 (13% for aged 6-18) for mental health disorders<br>157,050 (16% for aged 6-18) for neurotic disorders<br>322,478 (21% for aged 6-18) for self-inflicted injury/suicide
Analytic model	Poisson regression<br>Random-forest models
Covariate(s)	Holidays, day of week
Other adjustment	Seasonal/long-term trends using a natural spline smoothing function of time, measured in days with 2 degrees of freedom per season
Outcome	
Definition	Emergency department visits for mental health disorders (ICD-9: 290-319)<br>Emergency department visits for neurotic disorders (ICD-9 300–316)<br>Emergency department visits for self-inflicted injury/suicide (ICD9: E950-E959)
Data source	California Office of Statewide Health Planning and Development
Exposure	
Definition	Daily mean temperature at climate-zone level (n=16) measured using data from meteorological monitoring stations weighted by distance from monitor and population during summertime (May-Oct).
Lag structure	lag0
Data source	Environmental Protection Agency via the Air Quality System Data Mart, the California Irrigation Management Information System, and the National Oceanic and Atmospheric Administration.
Exclusion criteria	Excluded data on the last month (to avoid inclusion of persons admitted to the emergency department in 2013 who were not discharged until 2014).
Main findings	Each 5.6°C (10°F) increase in temperature (lag 0) was associated with 7.3% (4.0, 10.8), 9.5% (5.8, 13.3), and 6.7% (4.5, 8.9) higher risks of emergency department visits for mental health disorders, neurotic disorders and self-inflicted injury/suicide, respectively.
Conflicts of interest	None declared.

Study characteristics of Bernstein *et al.,* 2022

Characteristics	Details
Study design	Time-series
Study year	2016-2018
Geographical setting	US
Sex	Both
Age (year)	0-18
Sample size	69,995 for mental health disorders<br>33,229 for suicidality and depression
Analytic model	DLNM with quasi-Poisson distribution<br>Random-effects meta-analytic model
Covariate(s)	Relative humidity, federal holidays, and day of week
Other adjustment	Long-term trend: a natural cubic B-spline of time with nearly 1 df for every 10 years<br>Seasonal trend adjustment: a natural cubic B-spline calculated in days during warm season with 4 df for each year
Outcome	
Definition	Emergency department visits for mental health disorders (ICD-10-CM: F00-F99)<br>Emergency department visits for suicidality and depression (ICD-10-CM: R45.85, R45.86, R45.87, R45.1, R45.4, R45.5, R45.6, F32, F33)
Data source	Pediatric Health Information System
Exposure	
Definition	Daily maximum temperature at county-level measured using Parameter-elevation Relationships on Independent Slopes Model (PRISM) grid data (4-km) weighted by population during summertime (May-Sep).
Lag structure	lag07
Data source	National weather service
Exclusion criteria	-
Main findings	Days of extreme heat (95th pc) as compared with minimum morbidity temperature (MMT) (lag07) were associated with higher risks for emergency department visits for mental health disorders (RR=1.13, 0.97, 1.31), and suicidality and depression (RR=1.02, 0.93, 1.11).
Conflicts of interest	*"G.A.W. serves as a consultant to the Health Effects Institute (Boston, Massachusetts) and Google, LLC (Mountain View, California). All other authors declare they have no actual or potential competing financial interests"*

Study characteristics of Chan *et al.,* 2018

Characteristics	Details
Study design	Time-series
Study year	2016-2018
Geographical setting	Hong Kong
Sex	Both
Age (year)	<15
Sample size	833
Analytic model	Generalized additive model<br>DLNM
Covariate(s)	Daily mean relative humidity, holiday effect, day of week, same-day rainfall
Other adjustment	Long term trend (day of study), seasonal trend (day of year)
Outcome	
Definition	Mental health disorder hospitalizations (ICD-9: 290.xx-319.xx)
Data source	Hospital Authority of Hong Kong
Exposure	
Definition	Daily mean temperature at city-level measured using data from a meteorological monitoring station.
Lag structure	lag02
Data source	Hong Kong Observatory
Exclusion criteria	-
Main findings	The RR of mental health disorder hospitalizations associated with daily mean temperature (lag02) at 28°C (4th quartile) in reference to 19.4°C (lower quartile) was 0.99 (0.67, 1.47).
Conflicts of interest	None declared.

Study characteristics of Cohen *et al.,* 2024

Characteristics	Details
Study design	Case-crossover (matched each case with 3 or 4 control days on the same day of a week within the same month)
Study year	1995-2014
Geographical setting	New York, US
Sex	Both
Age (year)	≤24
Sample size	811,439 for mood disorders<br>447,286 for anxiety disorders<br>134,839 for adjustment disorders<br>27,888 for schizophrenia and other psychotic disorders
Analytic model	Conditional logistic regression<br>DLNM
Covariate(s)	daily mean temperature
Other adjustment	-
Outcome	
Definition	Hospital visits for mood disorders (ICD-9-CM: 293.83, 296, 300.4, 311.0)<br>Hospital visits for anxiety disorders (293.84, 300, 300.10, 300.2, 300.3, 300.5, 300.89, 300.9, 308.0, 308.1-308.4, 308.9, 309.81, 313.0-313.1, 313.21-313.22, 313.3, 313.82, 313.83)<br>Hospital visits for adjustments disorders (309.0, 309.1, 309.22-309.24, 309.28, 309.29, 309.3, 309.4, 309.82, 309.83, 309.89, 309.9)<br>Hospital visits for schizophrenia and other psychotic disorders (293.8, 295, 297, 298)
Data source	New York Department of Health Statewide Planning and Research Cooperative System (SPARCS)
Exposure	
Definition	Daily temperature range (difference between maximum and minimum temperatures) at ZIP code level measured using North American Land Data Assimilation System (NLDAS-2 Forcing) grid data (0.125° or 11-km*14km) weighted by population.
Lag structure	lag06
Data source	North American Land Data Assimilation System
Exclusion criteria	
Main findings	Higher daily temperature range (1st-99th percentile) in reference to minimum daily temperature range (0.1°C) was associated with a higher risk of hospital visits for mood disorders, anxiety disorders and adjustment disorders (effect estimates not shown).<br>Higher daily temperature range (5.2°C; 25th percentile) (0–6 days after exposure) in reference to minimum daily temperature range (0.1°C) was associated with a higher risk of hospital visits for schizophrenia [49.1%, (26.8, 75.3)].
Conflicts of interest	None declared.

Study characteristics of da Silva *et al.,* 2020

Characteristics	Details
Study design	Time-series
Study year	2010-2016
Geographical setting	Curitiba City, Brazil
Sex	Both
Age (year)	0-24
Sample size	Unknown (5,397 for population aged 0 to more than 60 years)
Analytic model	Generalized additive model<br>DLNM
Covariate(s)	Relative humidity, pollutant concentrations, day of week, and holidays
Other adjustment	A natural cubic spline with 7 df per year (temporal trend)
Outcome	
Definition	Mental health disorder hospitalizations (ICD-10: F00-F99)
Data source	Single-system of Health, Department of Informatics
Exposure	
Definition	Daily mean temperature at city-level measured using data from 3 meteorological monitoring stations.
Lag structure	lag0, lag 01, lag07
Data source	Environmental Institute of Paraná
Exclusion criteria	-
Main findings	The RRs of mental health disorder hospitalizations associated with daily mean temperature (lag 0), in reference to 22.4°C (mean), for men and women were 1.1400 (1.0551, 1.2366) and 1.1987 (1.1191, 1.2892), respectively.
Conflicts of interest	None declared.

Study characteristics of He *et al.,* 2025

Characteristics	Details
Study design	Case-crossover (matched each case with 3 or 4 control days on the same day of a week within the same month)
Study year	2001-2020
Geographical setting	New South Wales, Australia
Sex	Both
Age (year)	0-18
Sample size	91,938 daily emergency department visits for mental disorders<br>36,241 daily emergency department visits for suicidality and depression<br>30,017 daily hospital admissions for mental disorders<br>8,114 daily hospital admissions for suicidality and depression
Analytic model	Quasi-poisson model<br>DLNM
Covariate(s)	-
Other adjustment	-
Outcome	
Definition	Daily emergency department visits for mental disorders (ICD-10-AM: F00-F99)<br>Daily emergency department visits for suicidality and depression (ICD-10-AM: R45.85, R45.86, R45.87, R45.1, R45.4, R45.5, R45.6, F32, F33)<br>Daily hospital admissions for mental disorders (ICD-10-AM: F00-F99)<br>Daily hospital admissions for suicidality and depression (ICD-10-AM: R45.85, R45.86, R45.87, R45.1, R45.4, R45.5, R45.6, F32, F33)
Data source	The emergency department visit data were ascertained from the NSW Emergency Department Data Collection, whereas the hospital admission data were ascertained from the NSW Admitted Patient Data Collection.
Exposure	
Definition	Temperature using the ERA5 dataset at a spatial resolution of 0.25° × 0.25°<br>**A heatwave was defined as 2 consecutive days or more with daily maximum Universal Thermal Climate Index (UTCI) in the 95th percentile or higher.*
Lag structure	lag03
Data source	European Centre for Medium-Range Weather Forecast Reanalysis v5 Heat
Exclusion criteria	Encounters with missing information on residential address, age, or sex (n = 11 741 for ED visits and n = 4836 for admissions) were excluded.
Main findings	The RRs of emergency department visits for heatwave days as compared with non-heatwave days were 0.97 (0.91, 1.04) for mental disorders and 0.95 (0.86, 1.05) for suicidality. The RRs of hospital admission for heatwave days as compared with non-heatwave days were 1.14 (1.03, 1.26) for mental disorders and 1.16 (0.96, 1.40) for suicidality.
Conflicts of interest	*"The authors declare that they have no known competing financial interests or personal relationships that could have appeared to influence the work reported in this paper."*

Study characteristics of Hu *et al.,* 2025

Characteristics	Details
Study design	Cross-sectional
Study year	2021
Geographical setting	China
Sex	Both
Age (year)	10-18
Sample size	19,852
Analytic model	Logistic regression model
Covariate(s)	Grade, sex, region of school, family structure, parents' education, family income, physical activity, smoking, alcohol use, average precipitation
Other adjustment	-
Outcome	
Definition	Depression measured using the 9-item Patient Health Questionnaire-9 (PHQ-9) and defined using the threshold of 10<br>Anxiety measured using the 7-item Generalized Anxiety Disorder (GAD-7) and defined using the threshold of 10
Data source	Chinese adolescent health survey (CAHS)
Exposure	
Definition	Temperature measured using the ERA5-Land dataset at a spatial resolution of 0.1° × 0.1°.<br>**A heatwave was defined as period of three or more consecutive days with excess heat factor (EHF) values ≥0 over the 12 months prior to each survey time*
Lag structure	-
Data source	Fifth generation European ReAnalysis-Land (ERA5-Land)
Exclusion criteria	Excluded students who had severe mental disorders or to be taking psychiatric medication as reported by teachers or guardians, 48 participants with manifestly fictitious or inconsistent responses, and 253 participants who did not complete the questionnaire.
Main findings	Each additional heatwave day increment was associated with higher odds of depression (OR=1.13, 95% CI=1.09, 1.17) and anxiety (OR=1.12, 95% CI=1.08, 1.16).
Conflicts of interest	*"The authors declare that they have no known competing financial interests or personal relationships that could have appeared to influence the work reported in this paper."*

Study characteristics of Isaksen *et al.,* 2016

Characteristics	Details
Study design	Time-series
Study year	1980-2010
Geographical setting	King county, Washington, US
Sex	Both
Age (year)	0-14
Sample size	78,525 in 1980;<br>120,294 in 2010 (0-4)<br>170,657 in 1980;<br>224,084 in 2010 (5-14)
Analytic model	Poisson model (relative risk analysis)<br>Piecewise linear model (time series analysis)<br>DLNM
Covariate(s)	Age, gender, race, high school graduation, marital status, Hispanic origin, and tobacco use
Other adjustment	A natural cubic spline (df=19 over 31 years) (temporal trend)
Outcome	
Definition	Mortality associated with mental health disorders (ICD-9: 290-316 & ICD-10: F01-F69)
Data source	Washington State department of Health mortality dataset
Exposure	
Definition	Daily maximum temperature at county-level measured using Parameter-elevation Relationships on Independent Slopes Model (PRISM) grid data (1/16°) during summertime (May-Sep).
Lag structure	lag0
Data source	Weather station data derived from GHCN
Exclusion criteria	-
Main findings	The RRs of mortality associated with mental health disorders on a heat day (99th pc; lag 0) compared with a non-heat day were 0.99 (0.82, 1.19) for subgroup aged 0-4, and 1 (0.83, 1.2) for subgroup aged 5-14, respectively.
Conflicts of interest	None declared.

Study characteristics of Kim *et al.,* 2015

Characteristics	Details
Study design	Case-crossover (matched each case with 7 control days on the same day of the week over a 56-day time window)
Study year	1992-2010 for Korea;<br>1972-2010 for Japan;<br>1994-2007 for Taiwan
Geographical setting	Korea (Seoul, Busan, Incheon, Daegu, Daejeon, Gwangju);<br>Japan (Sapporo, Sendai, Tokyo, Nagoya, Osaka, Fukuoka);<br>Taiwan (Taipei, Taichung, Kaohsiung)
Sex	Both
Age (year)	10-24
Sample size	Unknown<br>(n=66,024 in Korea; n=126,705 in Japan; n=17,879 in Taiwan for population aged ≥10)
Analytic model	Conditional logistic regression
Covariate(s)	Sunshine duration, relative humidity, atmospheric pressure, date of suicide, and month
Other adjustment	-
Outcome	
Definition	Suicide deaths (ICD-8 & ICD-9: E950.0-E958.9; ICD-10: X60-X84)
Data source	Korea: Statistics Korea, Ministry of Strategy and Finance;<br>Japan: Ministry of Health, Labour and Welfare;<br>Taiwan: Department of Statistics, Ministry of Health and Welfare
Exposure	
Definition	Daily mean temperature (lag 0) at city-level measured using data from meteorological monitoring stations.
Lag structure	lag0
Data source	Korea: Korea Meteorological Administration;<br>Japan: Japan Meteorological Agency;<br>Taiwan: Taiwan Central Weather Bureau
Exclusion criteria	Excluding cases < 10 years of age
Main findings	Each 5.1°C, 4.0°C, 4.8°C, 4.7°C, 4.9°C and 4.6°C increment in temperature in Seoul, Busan, Incheon, Daegu, Daejeon and Gwangju was associated with 3.5% (-1.3, 8.5), 11.6% (3.5, 20.3), 3.4% (-6.0, 13.7), 6.1% (-3.4, 16.5), 15.7% (2.1, 31.0) and 12% (-0.8, 26.4) increments in suicides, respectively.<br>Each 4.8°C, 4.1°C, 3.9°C, 4.2°C, 4.1°C and 3.9°C increment in temperature in Sapporo, Sendai, Tokyo, Nagoya, Osaka and Fukuoka was associated with 1.7% (-6.8, 11.0), 5.9% (-5.8, 19.1), 4.5% (0.4, 8.7), 4.1% (-4.2, 13.1), 6.4% (-0.6, 13.9) and 13.2% (2.2, 25.4) increments in suicides, respectively.<br>Each 2.6°C, 2.4°C and 1.9°C increment in temperature in Taipei, Taichung and Kaohsiung was associated with 5.0% (-3.1, 13.8), 4.6% (-8.7, 19.9) and 6.1% (-0.5, 18.5) increments in suicides, respectively.
Conflicts of interest	None declared.

Study characteristics of Komulainen *et al.,* 2022

Characteristics	Details
Study design	Cohort (longitudinal)
Study year	From 1990-1995 to December 31, 2017
Geographical setting	Finland
Sex	Both
Age (year)	From age 10 until December 31, 2017
Sample size	365,482
Analytic model	Cox proportional hazards model
Covariate(s)	Sex, year of birth, month of birth, calendar year period, parental education, parental income, parental history of mental disorders, degree of urbanicity, and area-level socioeconomic characteristics (proportion of people without upper secondary education or higher, and proportion of people unemployed).
Other adjustment	-
Outcome	
Definition	Hospital admission (ICD-10: F20) or emergency unit visits or receiving outpatient care for schizophrenia
Data source	Care register for Health Care
Exposure	
Definition	Daily mean temperature from birth to 10th birthday at residential zip code level measured using gridded data (10km).
Lag structure	-
Data source	Finnish Meteorological Institute
Exclusion criteria	-
Main findings	Exposure to temperature in the highest quintile (Quintile 5), as compared with the lowest quintile, was associated with 10% higher risk of hospital admission for schizophrenia (HR=1.10, 95% CI=0.88, 1.37).
Conflicts of interest	None declared.

Study characteristics of Mullins & White 2019

Characteristics	Details
Study design	Time-series
Study year	2005-2016 for mental health disorders<br>1960-2016 for suicide deaths
Geographical setting	California, US for mental health disorders;<br>US for suicide deaths
Sex	Both
Age (year)	18-24
Sample size	8,294 for mental health disorders<br>2,096,460 for suicide deaths
Analytic model	Panel fixed effects method
Covariate(s)	County-by-month fixed effect, county/state-by-year fixed effect, and precipitation
Other adjustment	-
Outcome	
Definition	Emergency department visits for mental health disorders (ICD9-CM: 290-319; ICD-10: 'F' codes)<br>Suicide deaths (39-Cause code: 40; 34-Cause code: 350; ICD-7: 963, 970-979)
Data source	California's Office of Statewide Health Planning and Development & National Vital Statistics System
Exposure	
Definition	Daily mean temperature at county-level measured using Parameter-elevation Relationships on Independent Slopes Model (PRISM) grid (2.5 mile) weighted by inverse of square distance between the grid and population centroid of the county.
Lag structure	lag month
Data source	PRISM Climate Groups
Exclusion criteria	Inpatient visits that are often scheduled (e.g., surgery), or inevitable visits (e.g., childbirth) were excluded.
Main findings	Each 1°F increment in mean monthly temperature was associated with 0.72% (standard deviation=0.25) increase in monthly emergency department visits for mental health disorders.<br>Each 1°F increment in mean monthly temperature was associated with 0.33% (0.07) increase in monthly suicide rate.
Conflicts of interest	None declared.

Study characteristics of Ndovu *et al.,* 2025

Characteristics	Details
Study design	Case-crossover (matched each case with 3 or 4 control days on the same day of a week within the same month)
Study year	2005-2019
Geographical setting	California
Sex	Both
Age (year)	<19
Sample size	512,977 for mental disorders<br>163,187 for suicidality and depression
Analytic model	Conditional logistic regression<br>DLNM
Covariate(s)	-
Other adjustment	-
Outcome	
Definition	Emergency department visits and any unscheduled hospital admissions for mental health disorders (ICD-9: 290-319; ICD-10: F00-F99)<br>Emergency department visits and any unscheduled hospital admissions for suicidality and depression (ICD-9: 296.2-296.3, 311, V62.84; ICD-10: R45.8, F32-F33, X60-X84)
Data source	California Department of Health Care Access and Information
Exposure	
Definition	Daily maximum temperature at ZIP-code-level measured using Parameter-elevation Relationships on Independent Slopes Model (PRISM) grid (4-Km) weighted by inverse of square distance between the grid and population centroid.<br>*A heatwave was defined as days when the daily maximum temperature exceeded the 95th percentile of a year's daily maximum warm-season (May-Sep) temperature distribution in a ZIP code for ≥1 day
Lag structure	-
Data source	Gridded Surface Meteorological (gridMET) dataset
Exclusion criteria	Excluded patients less than 1 year old at the time of their visit (sensitivity analysis)
Main findings	Extreme heat events (95th percentile) were associated with increased Emergency department visits and any unscheduled hospital admissions for mental health disorders (OR=1.01, 95% CI=1.00, 1.03) and suicidality and depression (OR=1.01, 95% CI=0.99, 1.04)
Conflicts of interest	*"The authors declare they have no conflicts of interest related to this work to disclose."*

Study characteristics of Ngu *et al.,* 2017

Characteristics	Details
Study design	Ecological
Study year	1979-2016
Geographical setting	Sixty countries (excluding US, China, India and Russia)
Sex	Both
Age (year)	5-24
Sample size	16 countries (female; 5-14)<br>9 countries (female; 15-24)<br>10 countries (male; 5-14)<br>8 countries (male; 15-24)
Analytic model	Negative binomial models
Covariate(s)	-
Other adjustment	-
Outcome	
Definition	Suicide defined as fatal intentional self-harm derived from the WHO's mortality database.
Data source	WHO's mortality database
Exposure	
Definition	Temperature at country-level measured using fifth generation ECMWF atmospheric reanalysis of the global climate (ERA5) data.
Lag structure	-
Data source	ERA5 temperature data provided by European Centre for Medium-Range Weather Forecasts
Exclusion criteria	Excluded any countries without complete data for at least two thirds of the total timeframe
Main findings	One unit increase in heatwaves count was associated with 6.8% (IRR=1.068) increment of suicides and 6% (0.940) decrement of suicides.<br>One unit increase in heatwaves count was associated with 2.7% (1.027) increment of suicides and 3.5% (0.965) decrement of suicides.<br>One unit increase in heatwaves count was associated with 6.1% (0.939) decrement in suicide.<br>One unit increase in heatwaves count was associated with 2.3% (1.023) increment of suicides and 3% (0.970) decrement of suicides.
Conflicts of interest	None declared.

Study characteristics of Nitschke *et al.,* 2007

Characteristics	Details
Study design	Time-series
Study year	1993-2006
Geographical setting	Adelaide, Australia
Sex	Both
Age (year)	0-4 and 5-14
Sample size	-
Analytic model	Poisson regression model
Covariate(s)	Time trend implicitly adjusted for by design.<br>Seasonality was controlled for by excluding autumn and winter.
Other adjustment	-
Outcome	
Definition	Hospital admission for mental diseases (ICD9: 290-294-9, 580-5999; ICD-10: N00-N39)
Data source	South Australian Ambulance Service
Exposure	
Definition	Daily maximum temperature ≥35°C for ≥3 consecutive days (heatwave episodes) during spring and summer.
Lag structure	lag0
Data source	Bureau of Meteorology Kent Town
Exclusion criteria	
Main findings	During heatwaves in reference to non-heatwave periods, rates of daily events for hospital admission for mental diseases increased by 52% (IRR=1.52, 0.99-2.32) and 9% (IRR=1.09; 0.85-1.39) for individuals aged 0-4 and 5-14 years, respectively.
Conflicts of interest	None declared.

Study characteristics of Niu *et al.,* 2020

Characteristics	Details
Study design	Time-series
Study year	2016-2018
Geographical setting	Beijing, China
Sex	Both
Age (year)	<18
Sample size	1,653
Analytic model	Poisson generalized additive model<br>DLNM
Covariate(s)	Sunshine duration, precipitation, $PM_{2.5}$, SO_2, O_3, day of week, holiday
Other adjustment	A natural cubic spline with 4 df per year
Outcome	
Definition	Emergency admissions associated with mental health disorders (ICD-10: F00-F99)
Data source	Beijing Municipal Health Commission Information Center
Exposure	
Definition	Daily mean temperature at city-level measured using data from 3 meteorological monitoring stations weighted by water vapor pressure and wind velocity.
Lag structure	- lag0, lag1, lag2…lag7<br>- lag0, lag01, lag02…lag07
Data source	China Meteorological Data Service Center
Exclusion criteria	Excluded patients for return visits
Main findings	The RRs of emergency visits for mental health disorders associated with high temperature (9.2°C; 90th pc) at lag 0 and lag 1, in reference to -2.4°C were 0.994 (0.404-2.447) and 1.154 (0.772-1.727), respectively.
Conflicts of interest	None declared.

Study characteristics of Niu *et al.,* 2023

Characteristics	Details
Study design	Case-crossover (matched each case with 3 or 4 control days on the same day of a week within the same month)
Study year	2005-2011
Geographical setting	New York City, US
Sex	Both
Age (year)	6-17
Sample size	82,982
Analytic model	Conditional logistic regression
Covariate(s)	Relative humidity (for sensitivity test)
Other adjustment	-
Outcome	
Definition	Emergency department visits and hospitalizations for mental health disorders (ICD-9: 290-299);<br>Anxiety (300-300.3, 300.5-300.9, 309.21, 309.81);<br>Depression (296.2-296.39, 311, 300.4, 296.9-296.99);<br>Suicide and self-inflicted injury (E95);<br>Externalizing disorders (312-313.82);<br>Reaction disorders (308-309.2, 309.22-309.8, 309.82-309.9);<br>Psychosis (290–295.95, 297–298.9);<br>Bipolar disorder (296-296.16, 296.4-296.89);<br>Substance abuse (303-305.93)
Data source	New York Statewide Planning and Research Cooperative System
Exposure	
Definition	Daily minimum temperature at city-level measured using data from 4 meteorological monitoring stations during summertime (Jun-Aug).
Lag structure	lag05
Data source	NOAA NCDC
Exclusion criteria	Excluded cases with another cause as primary diagnosis and a mental health diagnosis as secondary diagnosis
Main findings	An increased temperature (76°F, 95th pc) in reference to MRT (69.2°F, 65.2°F, and 58.7°F) was associated with a higher odds of mental health disorders for children (OR=1.28, 1.13, 1.46), and adolescents (1.17, 1.09, 1.25).<br>An increased temperature (95th pc) in reference to MRT (50.5°F, 63.1°F, and 82.7°F) was associated with a higher odds of anxiety disorder for children (1.66, 0.45, 6.12), and adolescents (1.42, 1.08, 1.87).<br>An increased temperature (95th pc) in reference to MRT (69.5°F, 66.8°F, and 82.7°F) was associated with a higher odds of depression for children (1.31, 0.83, 2.07), and adolescents (1.13, 0.94, 1.36).<br>An increased temperature (95th pc) in reference to MRT (65.8°F, and 50.5°F) was associated with a higher odds of suicide/self-inflicted injury for adolescents (1.19, 0.85, 1.67).<br>An increased temperature (95th pc) in reference to MRT (69°F, 65.6°F, and 50.5°F) was associated with a higher odds of externalizing disorders for children (1.20, 0.86, 1.68), and adolescents (1.08, 0.90, 1.30).

	An increased temperature (95th pc) in reference to MRT (69.7°F, 63.8°F, and 58.5°F) was associated with a higher odds of reaction disorder for children (2.21, 1.22, 3.99), and adolescents (1.23, 0.9, 1.68).<br>An increased temperature (95th pc) in reference to MRT (57.5°F, and 65.1°F) was associated with a higher odds of psychosis for adolescents (1.34, 0.93, 1.94).<br>An increased temperature (95th pc) in reference to MRT (69.6°F, and 50.5°F) was associated with a higher odds of bipolar disorder for adolescents (1.39, 1.06, 1.83).<br>An increased temperature (95th pc) in reference to MRT (82.7°F, and 50.5°F) was associated with a higher odds of substance abuse for adolescents (1.06, 0.66, 1.72).
Conflicts of interest	None declared.

Abbreviation: MRT, minimum risk temperature

Study characteristics of Parks *et al.,* 2020

Characteristics	Details
Study design	Time-series
Study year	1980-2017
Geographical setting	US
Sex	Both
Age (year)	5-14
Sample size	7,748 boys & 2,971 girls
Analytic model	Bayesian spatio-temporal model
Covariate(s)	State, month of year overall time (in months)
Other adjustment	-
Outcome	
Definition	Suicide deaths (ICD-9: E950-E959 & ICD-10: X60-X84)
Data source	National Center for Health Statistics
Exposure	
Definition	Monthly temperature at state-level using ERA5 grid data (30-km) weighted by population.
Lag structure	-
Data source	ERA5
Exclusion criteria	Excluded Alaska and Hawaii
Main findings	Suicide deaths were estimated to increase for a 1.5°C increase in anomaly for both men and women (data not shown).
Conflicts of interest	*"M.E. reports a charitable grant from AstraZeneca Young Health Programme, and personal fees from Prudential, Scor and Third Bridge, all outside the submitted work; all other authors declare no competing interests."*

Study characteristics of Rahman *et al.,* 2023

Characteristics	Details
Study design	Case-crossover (matched each case with 3 or 4 control days on the same day of a week within the same month)
Study year	2014-2019
Geographical setting	California, US
Sex	Both
Age (year)	≤24
Sample size	2,875
Analytic model	Conditional logistic regression<br>DLNM
Covariate(s)	Same lag day relative humidity
Other adjustment	-
Outcome	
Definition	Suicide deaths (ICD-10: X60-X84, Y87.0)
Data source	California Department of Public Health
Exposure	
Definition	Daily minimum and maximum temperatures at census tract level measured using gridMET data (4-km).
Lag structure	lag0, lag1, lag2…lag7
Data source	gridMET
Exclusion criteria	-
Main findings	The ORs per 1°C increase in maximum temperature (lag 0) was 0.994 (0.984, 1.005), while that for minimum temperature (lag 0) was 0.997 (0.981, 1.014).
Conflicts of interest	None declared.

Study characteristics of Runkle *et al.,* 2025

Characteristics	Details
Study design	Retrospective matched-case (heatwave days being designated as case days each matched with three reference non-heatwave days that meet 4 criteria: (1) same county as case day; (2) same month as case day; (3) do not coincide with or fall within 3 days of a heatwave event; (4) do not occur in the same year as the case)
Study year	2008-2021
Geographical setting	North Carolina
Sex	Both
Age (year)	5-24
Sample size	1,029 for major depressive disorder<br>3,712 for suicidal behavior
Analytic model	Poisson mixed-effect regression models
Covariate(s)	Population size
Other adjustment	A county-specific random intercept and fixed effects for day of week, year, and a binary indicator variable to control for warm season trends (May through Sep)
Outcome	
Definition	Daily emergency department visits for major depressive disorder (ICD-9: 296.3; ICD-10: F33)<br>Daily emergency department visits for suicidal behavior (ICD-9: E95, V628.4; ICD-10: X60-X84, R45.851, T14.91)
Data source	University of North Carolina Cecil G. Sheps Center for Health Services Research
Exposure	
Definition	Daily mean temperature at county-level during summertime (May-Sep) measured using the nClimGrid data at a resolution of 1/24 of a degree (0.0417°).<br>**A heatwave day was defined as the Excess Heat Factor (EHF) >0. The EHF combines two metrics: (1) EHF Significance Index (EHFsig), comparing the current 3-day mean temperature to the long-term 95th percentile threshold, and (2) EHF Acclimatization Index (EHFaccl), calculated as the deviation from the preceding 30-day mean.*
Lag structure	- lag0, lag1, lag2…lag7<br>- lag03, lag05, lag07
Data source	NOAA's nClimGrid product
Exclusion criteria	-
Main findings	Days of heatwave days as compared with matched non-heatwave days at lag 0 was associated with higher risk of emergency department visits for major depressive disorder (RR=1.17, 95% CI=1.01, 1.34) and suicide behavour (RR=1.03, 0.96, 1.12).
Conflicts of interest	*"The authors have declared that no competing interests exist"*

Study characteristics of Stowell *et al.,* 2023

Characteristics	Details
Study design	Case-crossover (matched each case period with control period within the same year, month, and day of the week)
Study year	2016-2019
Geographical setting	US
Sex	Both
Age (year)	0-17
Sample size	26,366 for mental, behavioural disroders;<br>16,579 for suicidality and depression
Analytic model	Conditional logistic regression model<br>DLNM
Covariate(s)	Quadratic B-spline with one internal knot placed at 50th percentile (exposure-response functions)<br>Natural cubic B-spline with two knots placed at equal intervals on the log scale of lags up to 5 days (lag response function)<br>Natural spline function with 3df for daily mean relative humidity and federal holidays
Other adjustment	-
Outcome	
Definition	Emergency department visits for mental, behavioural disroders (ICD-10: F00-F99)<br>Emergency department visits for suicidality and depression (ICD-10: R45.85-R45.87, R45.1, R45.4-R45.6, F32, F33)
Data source	Optum Labs Data Warehouse
Exposure	
Definition	Daily maximum temperature at county-level measured using Parameter-elevation Relationships on Independent Slopes Model (PRISM) grid data (4-km) weighted by population during summertime (May-Sep).
Lag structure	lag05
Data source	National weather service
Exclusion criteria	Excluded county-days with >20% missing data
Main findings	A day of extreme heat (95th pc) as compared with 50th percentile of the daily temperature was associated with higher rates of ED visits for mental, behavioural disroders (OR=1.01, 95% CI=0.93, 1.10) and suicidality and depression (OR=1.04, 95% CI=0.93, 1.15).
Conflicts of interest	*"Dr Wellenius serves as a consultant for Google, LLC (Mountain View, CA) and the Health Effects Institute (Boston, MA). The authors declare that they have no known competing financial interests or personal relationships that could have influenced the work reported in this paper."*

Study characteristics of Trang *et al.,* 2016

Characteristics	Details
Study design	Time-series
Study year	2008-2012
Geographical setting	Hanoi, Vietnam
Sex	Both
Age (year)	0-17
Sample size	655
Analytic model	Zero-inflated negative binomial regression model
Covariate(s)	Day of the week, season, and time trend (natural cubic spline function, 16 df)
Other adjustment	-
Outcome	
Definition	Hospital admission for mental health disorder (ICD-10: F00-F99, except F60-F69)
Data source	Hanoi Mental Hospital
Exposure	
Definition	Yearly average of maximum temperature at postcode level measured using gridded data (1km).
Lag structure	lag 0, lag03, lag07
Data source	Average maximum temperature derived from several meteorological monitoring stations
Exclusion criteria	-
Main findings	Heatwaves (daily maximum temperature exceeding 35°C for one day or ⩾3 or ⩾7 consecutive days) were associated with higher risks of mental disorders (insignificant; data not shown).
Conflicts of interest	None declared.

Study characteristics of Villeneuve *et al.,* 2023

Characteristics	Details
Study design	Case-crossover (matched each case with 3 or 4 control days on the same day of a week within the same month)
Study year	2002-2015
Geographical setting	Canada
Sex	Both
Age (year)	<25
Sample size	6,813
Analytic model	Conditional logistic model
Covariate(s)	Holidays (main)<br>Relative humidity and precipitation and air pollutants (sensitivity test)
Other adjustment	-
Outcome	
Definition	Suicide deaths (ICD-10: X60-X84)
Data source	Canadian Vital Statistics Death Database
Exposure	
Definition	Daily mean temperature at postal code level measured using interpolated metrics weighted by daily count of suicides deaths.
Lag structure	lag0, lag1, lag2<br>lag01, lag02
Data source	Canadian Urban Environmental Health Research Consortium (CANUE)
Exclusion criteria	-
Main findings	An IQR (9.6°C) increase in daily mean temperature (lag 0) was associated with a higher odds of suicide death (1.097, 1.069, 1.126).
Conflicts of interest	None declared.

Study characteristics of Wang *et al.,* 2018

Characteristics	Details
Study design	Time-series
Study year	2005-2014
Geographical setting	Heifei, China
Sex	Both
Age (year)	≤20
Sample size	3,361
Analytic model	Poisson generalized linear regression model<br>DLNM
Covariate(s)	Rainfall, day of week and holiday
Other adjustment	Natural cubic spline with 3df per year
Outcome	
Definition	Hospital admission of schizophrenia (ICD-10: F20-F29)
Data source	Anhui Mental Health Centre
Exposure	
Definition	Daily mean temperature measured using data from meteorological station during summertime time (May-Oct).
Lag structure	lag0, lag1…lag6<br>lag0, lag01…lag06
Data source	Hefei Bureau of Meteorology
Exclusion criteria	-
Main findings	A high warm-season temperature (28°C; 75th pc) at lag 0 in reference to lower temperature (24.8°C; 50th pc) was associated with higher risk of schizophrenia (RR=1.03, 0.98, 1.09).
Conflicts of interest	None declared.

Study characteristics of Xu *et al.,* 2018

Characteristics	Details
Study design	Cohort (cross-sectional)
Study year	2008-2014
Geographical setting	Australia
Sex	Both
Age (year)	6-11
Sample size	14,096
Analytic model	Structural equation modelling
Covariate(s)	Regular participation in organised sport and other physical activities in the past 12 months, gender, age, household size, family income, religion and ethnicity.
Other adjustment	-
Outcome	
Definition	Mental health based on the Strengths and Difficulties Questionnaire (SDQ).
Data source	*Longitudinal Study of Australian Children*
Exposure	
Definition	Yearly average of maximum temperature at postcode level measured using gridded data (1km).
Lag structure	-
Data source	Australian Bureau of Meteorology
Exclusion criteria	-
Main findings	1 °C increase in annual mean of maximum temperature was associated with a 0.05 (0.02, 0.07) SDQ total score increase.
Conflicts of interest	None declared.

Study characteristics of Zhang *et al.,* 2020

Characteristics	Details
Study design	Case-crossover (matched each case with 3 or 4 control days on the same day of a week within the same month)
Study year	2016-2018 for Shenzhen<br>2016-2018 for Zhaoqing<br>2013-2018 for Huizhou
Geographical setting	Shenzhen, Zhaoqing, and Huizhou, China
Sex	Both
Age (year)	<18
Sample size	191,020 in Zhaoqing (4.4% for <18);<br>649,052 in Shenzhen (5.2% for <18);<br>293,148 in Huizhou (4.7% for <18)
Analytic model	Quasi-Poisson generalized linear model<br>DLNM
Covariate(s)	Air pollutants, relative humidity, day of week, public holiday
Other adjustment	A smoothing function of time calculated in years with a 7df per year
Outcome	
Definition	Hospital outpatient visits for depressive disorders (ICD-10: F32-F33), anxiety (F40-F41), organic mental health disorders (F00-F09), schizophrenia (F20-F29), & affective disorders (excluding F30-F31 & F34-F39).
Data source	Psychiatric specialist hospitals
Exposure	
Definition	Temperature using data from meteorological monitoring stations.
Lag structure	lag 0, lag01, lag03, lag05, lag07, lag09
Data source	Meteorological Data Sharing Center under the National Weather Service
Exclusion criteria	Residing outside of the three study cities more than six months and had missing information were excluded from the analysis.
Main findings	The RRs of depressive disorders, anxiety, organic mental health disorders, schizophrenia, and affective disorders associated with extreme hot temperature (30°C; 97.5th pc) (lag 09) in reference to temperature of minimum risk (18°C) were 1.04 (0.95, 1.14), 0.98 (0.59, 1.61), 1.17 (0.48, 2.85), 1.08 (0.99, 1.17), and 1.33 (0.78, 2.28) respectively.
Conflicts of interest	None declared.

Study characteristics of Zhong *et al.,* 2025

Characteristics	Details
Study design	Case-crossover (matched each case with 3 or 4 control days on the same day of a week within the same month)
Study year	2019-2021
Geographical setting	Anhui, China
Sex	Both
Age (year)	<18
Sample size	76,023 for mental disorders
Analytic model	Conditional logistic regression models<br>DLNM
Covariate(s)	Relative humidity, wind speed, precipitation, $PM_{2.5}$, holiday, COVID
Other adjustment	-
Outcome	
Definition	Outpatient visits for mental and behavioral disorders (ICD-10: F00-F99)<br>Outpatient visits for schizophrenia (ICD-10: F20-F21)<br>Outpatient visits for depression (ICD-10: F32-F33)<br>Outpatient visits for anxiety (ICD-10: F40-F41)
Data source	Anhui Mental Health Center
Exposure	
Definition	Daily mean temperature using the CN05.1 grid observation dataset at a spatial resolution of 0.25° × 0.25°
Lag structure	lag021
Data source	CN05.1 grid observation dataset
Exclusion criteria	-
Main findings	Exposure to heat extremes (both the daily maximum and minimum temperatures being equal to or higher than the 90th percentile temperature, duration ⩾1 day) was associated with higher odds of outpatient visits for mental disorders (OR=1.16, 95% CI=1.02, 1.31), schizophrenia (OR=1.12, 95% CI=0.72, 1.75), depression (OR=1.50, 95% CI=1.20, 1.88). A negative association was observed between heat extremes and anxiety (OR=0.89, 95% CI=0.60, 1.32).
Conflicts of interest	*"The authors declare that they have no known competing financial interests or personal relationships that could have appeared to influence the work reported in this paper."*

Study characteristics of Zhou *et al.,* 2023

Characteristics	Details
Study design	Time-series
Study year	2014-2019
Geographical setting	Chongqing, China
Sex	Both
Age (year)	≤18
Sample size	13,329
Analytic model	Quasi-Poisson generalized linear model<br>DLNM
Covariate(s)	Day of week, holiday, wind velocity and rainfall, O_3 and $PM_{2.5}$
Other adjustment	Calendar time (a natural cubic spline with 7 df per year)
Outcome	
Definition	Outpatient visits for depression (ICD-10: F32)
Data source	Xinqiao Hospital and Southwest Hospital
Exposure	
Definition	Temperature at city-level measured using data from a meteorological monitoring station.
Lag structure	lag 0, lag 1, lag 2…lag14<br>lag 0, lag01, lag 02…lag014
Data source	Chongqing Meteorological Bureau
Exclusion criteria	-
Main findings	The RR on the association between extremely high humidex (>40; lag 0) and depression outpatient visits was 0.961 (0.890, 1.038).
Conflicts of interest	None declared.

Study characteristics of Zhou *et al.,* 2024

Characteristics	Details
Study design	Time-series
Study year	2013-2020
Geographical setting	Liuzhou, China
Sex	Both
Age (year)	≤20
Sample size	966
Analytic model	Poisson generalized linear model<br>DLNM
Covariate(s)	Relative humidity, precipitation, day of week, and holiday
Other adjustment	A natural cubic spline with 8 df per year
Outcome	
Definition	Hospital admission for schizophrenia (ICD-10: F20-F20.9)
Data source	Guangxi Zhuang Autonomous Region Brain Hospital
Exposure	
Definition	Daily mean temperature measured using data from meteorological station.
Lag structure	lag 0, lag 1, lag 2…lag14<br>lag 0, lag01, lag 02…lag014
Data source	China Meteorological Data Service Centre
Exclusion criteria	Residential addresses outside of Liuzhou were excluded
Main findings	The RR on the association between high temperature (lag0) in reference to 21.7°C (75th pc) and hospital admission for schizophrenia was 1.04 (0.712, 1.519).
Conflicts of interest	None declared.

Supplementary File 10. Summary of risk of bias of individual studies as per the US National Toxicology Program's Office of Health Assessment and Translation (OHAT) risk of bias rating tool.

Study design	Lead author (year)	Confounding bias	Detection bias for exposure	Detection bias for outcome	Selective reporting bias	Other bias
Time-series	Basu *et al.*, 2018	+	+	+	+	+
	Bernstein *et al.*, 2022	+	+	+	+	+
	Chan *et al.*, 2018	+	-	+	+	+
	da Silva *et al.*, 2020	+	-	+	+	+
	Isaksen *et al.*, 2016	+	+	+	+	+
	Mullins & White, 2019	+	+	+	+	+
	Nitschke *et al.*, 2007	+	-	-	+	+
	Niu *et al.*, 2020	+	-	+	+	+
	Parks *et al.*, 2020	-	+	+	+	+
	Trang *et al.*, 2016	+	+	+	+	+
	Wang *et al.*, 2018	+	-	+	+	+
	Zhou *et al.*, 2023	+	-	+	+	+
	Zhou *et al.*, 2024	+	-	+	+	+
Case-crossover	Cohen *et al.*, 2024	+	+	+	+	+
	He *et al.*, 2025	+	+	+	+	+
	Kim *et al.*, 2016	+	-	+	+	+
	Ndovu *et al.*, 2025	+	+	+	+	+
	Niu *et al.*, 2023	+	-	+	+	+
	Rahman *et al.*, 2023	+	+	+	+	+
	Stowell *et al.*, 2023	+	+	+	+	+
	Villeneuve *et al.*, 2023	+	+	+	+	+
	Zhang *et al.*, 2020	+	-	+	+	+
	Zhong *et al.*, 2025	+	+	+	+	+
Cohort	Hu *et al.*, 2025	+	+	+	+	+

	Komulainen *et al.*, 2022	+	+	+	+	+
	Xu *et al.*, 2018	+	+	+	+	+
Retrospective case-matched	Runkle *et al.*, 2025	+	+	+	+	+
Ecological	Ngu *et al.*, 2021	-	+	+	+	-

Level of risk of bias

++	Definitely Low	+	Probably Low	- NR	Probably High/ Not reported	--	Definitely High

Supplementary File 11. Risk of bias summary for individual students.

Risk of bias summary for Basu *et al.*, 2018.

**Risk of bias domain**	**Author's judgement**	**Support for judgement**
Confounding bias	Probably Low	- Covariates included holidays, day of the week, and seasonal/long-term trends.
Detection bias for exposure	Probably Low	- Daily mean temperature measured from 401 meteorological monitoring stations during warm season (May-Oct). Temperature was calculated at the climate-zone level (n=16) combined from zip-code tabulation area (centroid)-level data.<br>- Same-day temperature (lag 0) was weighted by distance from each monitor and zip-code level population count.<br>- Data sourced from the Environmental Protection Agency via the Air Quality System Data Mart, the California Irrigation Management Information System, and the National Oceanic and Atmospheric Administration.
Detection bias for outcome	Probably Low	- Emergency department visits for all mental disorders (primary diagnosis) defined based on ICD-9 codes (290-319).<br>- Emergency department visits for self-inflicted injury/suicide (regardless of primary diagnosis) defined based on ICD-9 codes (E950-E959).<br>- Data sourced from the California Office of Statewide Health Planning and Development.
Selective reporting bias	Probably Low	- No selective reporting bias is suspected.
Other bias	Probably Low	- No other bias is suspected.

Risk of bias summary for Bernstein *et al.,* 2022.

**Risk of bias domain**	**Author's judgement**	**Support for judgement**
Confounding bias	Probably Low	- Covariates included temporal trends, seasonality, relative humidity, federal holidays, and day of the week.
Detection bias for exposure	Probably Low	- Daily maximum temperature measured at the county-level (based on hospital locations) using PRISM pixels across census tracts during warm season (May-Sep).<br>- Temperature was measured using a period-based system (lag 07 days) weighted by population.<br>- Data was sourced from the National weather service.
Detection bias for outcome	Probably Low	- Emergency department visits for mental, behavioral, and neurodevelopmental disorders (primary discharge diagnosis) defined based on ICD-10CM codes (F00-F99).<br>- Emergency department visits for suicidality and depression (primary discharge diagnosis) defined based on ICD-10-CM codes (R45.85, R45.86, R45.87, R45.1, R45.4, R45.5, R45.6, F32, F33)<br>- Data was sourced from Pediatric Health Information System.
Selective reporting bias	Probably Low	- No selective reporting bias is suspected.
Other bias	Probably Low	- No other bias is suspected.

Risk of bias summary for Chan *et al.,* 2018.

Risk of bias domain	Author's judgement	Support for judgement
Confounding bias	Probably Low	- Daily mean relative humidity, long term trend (day of study), seasonal trend, holiday effect, day-of-week effect, same-day rainfall
Detection bias for exposure	Probably High	- Daily mean temperature derived from a meteorological monitoring station<br>- Temperature was measured on the same day of event, and further measured based on a period-based system (lag02).<br>- Data sourced from the Hong Kong Observatory (located near center of Hong Kong)
Detection bias for outcome	Probably Low	- Mental disorder hospitalizations defined based on ICD-9 codes (290.xx-319.xx) (principal diagnosis at discharge)<br>- Data sourced from the Hospital Authority of Hong Kong
Selective reporting bias	Probably Low	- No selective reporting bias is suspected.
Other bias	Probably Low	- No other bias is suspected.

Risk of bias summary for Cohen *et al.,* 2024.

**Risk of bias domain**	**Author's judgement**	**Support for judgement**
Confounding bias	Probably Low	- Adjusted for mean temperature<br>- Case-crossover study: individual confounders and long-term trends are adjusted for by design.
Detection bias for exposure	Probably Low	- Daily temperature range (difference between maximum and minimum temperatures) at ZIP code Tabulation Area (ZCTA) level measured using NLDAS-2 Forcing grid data (0.125° or 11-km*14km) weighted by population.<br>- Temperature was measured at the ZIP code level calculated based on a period-based structure (lag 06).<br>- Data were sourced from the North American Land Data Assimilation System.
Detection bias for outcome	Probably Low	- Hospital visits for mood disorders (ICD-9-CM: 293.83, 296, 300.4, 311.0)<br>- Hospital visits for anxiety disorders (293.84, 300, 300.10, 300.2, 300.3, 300.5, 300.89, 300.9, 308.0, 308.1-308.4, 308.9, 309.81, 313.0-313.1, 313.21-313.22, 313.3, 313.82, 313.83)<br>- Hospital visits for adjustments disorders (309.0, 309.1, 309.22-309.24, 309.28, 309.29, 309.3, 309.4, 309.82, 309.83, 309.89, 309.9)<br>- Hospital visits for schizophrenia and other psychotic disorders (293.8, 295, 297, 298)<br>- Data sourced from the New York Department of Health Statewide Planning and Research<br>- Cooperative System (SPARCS).
Selective reporting bias	Probably Low	- No selective reporting bias is suspected.
Other bias	Probably Low	- No other bias is suspected.

Risk of bias summary for da Silva *et al.,* 2020.

**Risk of bias domain**	**Author's judgement**	**Support for judgement**
Confounding bias	Probably Low	- Covariates included relative humidity, pollutant concentrations, temporal trend, day of the week and holidays.
Detection bias for exposure	Probably High	- Daily mean temperature measured from three meteorological monitoring stations.<br>- Temperature was measured at the city-level on the same day of event onset (lag 0) and further calculated based on a period-based structure (lag 01, lag 07 days).<br>- Data sourced from Environmental Institute of Paraná.
Detection bias for outcome	Probably Low	- Daily number of hospitalizations for mental and behavioural disorders (primary diagnosis) defined based on ICD-10 codes (F00-F99).<br>- Data were sourced from Single-system of Health, Department of Informatics.
Selective reporting bias	Probably Low	- No selective reporting bias is suspected.
Other bias	Probably Low	- No other bias is suspected.

Risk of bias summary for He *et al.,* 2025.

**Risk of bias domain**	**Author's judgement**	**Support for judgement**
Confounding bias	Probably Low	- Case-crossover study: individual confounders and long-term trends are adjusted for by design.
Detection bias for exposure	Probably Low	- Temperature using the ERA5 dataset at a spatial resolution of 0.25° × 0.25°.<br>- Temperature measured over lag03.<br>- Data were sourced from the European Centre for Medium-Range Weather Forecast Reanalysis v5 Heat.
Detection bias for outcome	Probably Low	- Daily emergency department visits for mental disorders (ICD-10-AM: F00-F99)<br>- Daily emergency department visits for suicidality and depression (ICD-10-AM: R45.85, R45.86, R45.87, R45.1, R45.4, R45.5, R45.6, F32, F33)<br>- Daily hospital admissions for mental disorders (ICD-10-AM: F00-F99)<br>- Daily hospital admissions for suicidality and depression (ICD-10-AM: R45.85, R45.86, R45.87, R45.1, R45.4, R45.5, R45.6, F32, F33)<br>- The emergency department visit data were ascertained from the NSW Emergency Department Data Collection, whereas the hospital admission data were ascertained from the NSW Admitted Patient Data Collection.
Selective reporting bias	Probably Low	- No selective reporting bias is suspected.
Other bias	Probably Low	- No other bias is suspected.

Risk of bias summary for Hu *et al.*, 2025.

**Risk of bias domain**	**Author's judgement**	**Support for judgement**
Confounding bias	Probably Low	- Covariates included grade, sex, region of school, family structure, parents' education, family income, physical activity, smoking, alcohol use, average precipitation.
Detection bias for exposure	Probably Low	- Temperature measured using the ERA5-Land dataset at a spatial resolution of 0.1° × 0.1°.<br>- Data were sourced from the Fifth generation European ReAnalysis-Land (ERA5-Land).
Detection bias for outcome	Probably Low	- Depression measured using the 9-item Patient Health Questionnaire-9 (PHQ-9) and defined using the threshold of 10<br>- Anxiety measured using the 7-item Generalized Anxiety Disorder (GAD-7) and defined using the threshold of 10<br>- Data were sourced from the Chinese adolescent health survey (CAHS).
Selective reporting bias	Probably Low	- No selective reporting bias is suspected.
Other bias	Probably Low	- No other bias is suspected.

Risk of bias summary for Isaksen *et al.,* 2016.

**Risk of bias domain**	**Author's judgement**	**Support for judgement**
Confounding bias	Probably Low	- Adjusted for the seasonal monthly effects
Detection bias for exposure	Probably Low	- Daily maximum temperature derived from spatiotemporal models [(1/16° grid data using Parameter-elevation Relationships on Independent Slopes Model (PRISM)] during warm season (May-Sep).<br>- Temperature was measured at the county level on the same day of event (lag 0) accounting for effects of humidity.<br>- Weather station data derived from Global Historical Climate Network-Daily (GHCN).
Detection bias for outcome	Probably Low	- Mortality associated with mental disorders defined as per ICD-9 (290-316; year 1980-1998) and ICD-10 (F01-F69; year 1999-2010).<br>- Data sourced from the Washington State department of Health mortality dataset.
Selective reporting bias	Probably Low	- No selective reporting bias is suspected.
Other bias	Probably Low	- No other bias is suspected.

Risk of bias summary for Kim *et al.,* 2016.

**Risk of bias domain**	**Author's judgement**	**Support for judgement**
Confounding bias	Probably Low	- Covariates included sunshine duration, relative humidity, atmospheric pressure, date of suicide, and month.<br>- Case-crossover study: individual confounders and long-term trends are adjusted for by design.
Detection bias for exposure	Probably High	- Daily mean temperature derived from meteorological monitoring stations.<br>- Temperature was measured at the city-level on the same day of event (lag 0).<br>- Data were sourced from the Korea Meteorological Administration, Japan Meteorological Agency, and Taiwan Central Weather Bureau.
Detection bias for outcome	Probably Low	- Suicide deaths defined based on ICD-8, ICD-9 (E950.0-E958.9) and ICD-10 (X60-X84)<br>- Data were sourced from the Statistics Korea, Ministry of Strategy and Finance (Korea), Ministry of Health, Labour and Welfare (Japan), and the Department of Statistics, Ministry of Health and Welfare (Taiwan).
Selective reporting bias	Probably Low	- No selective reporting bias is suspected.
Other bias	Probably Low	- No other bias is suspected.

Risk of bias summary for Komulainen *et al.,* 2022.

**Risk of bias domain**	**Author's judgement**	**Support for judgement**
Confounding bias	Probably Low	- Covariates included sex, year of birth, month of birth, calendar year period, parental education, parental income, parental history of mental disorders, degree of urbanicity, and area-level socioeconomic characteristics (proportion of people without upper secondary education or higher, and proportion of people unemployed).
Detection bias for exposure	Probably Low	- Daily mean temperature from birth to 10th birthday at residential zip code level measured using gridded data (10km).<br>- Data were sourced from the Finnish Meteorological Institute.
Detection bias for outcome	Probably Low	- Hospital admission (ICD-10: F20) or emergency unit visits or receiving outpatient care for schizophrenia<br>- Data were sourced from the Care register for Health Care.
Selective reporting bias	Probably Low	- No selective reporting bias is suspected.
Other bias	Probably Low	- No other bias is suspected.

Risk of bias summary for Mullins & White 2019.

Risk of bias domain	Author's judgement	Support for judgement
Confounding bias	Probably Low	- Adjusted for county-by-month fixed effect, county/state-by-year fixed effect, and precipitation.
Detection bias for exposure	Probably Low	- Daily mean temperature data measured at the county-level derived from the PRISM grid data provided by PRISM Climate Groups.
Detection bias for outcome	Probably Low	- Emergency department visits for mental health disorders defined based on ICD9-CM: (290-319) and ICD-10: ('F' codes).<br>- Suicide deaths defined based on 39-Cause code (40) and 34-Cause code (350) and ICD-7 (963, 970-979).
Selective reporting bias	Probably Low	- No selective reporting bias is suspected.
Other bias	Probably Low	- No other bias is suspected.

Risk of bias summary for Ndovu *et al.,* 2025.

**Risk of bias domain**	**Author's judgement**	**Support for judgement**
Confounding bias	Probably Low	- Case-crossover study: individual confounders and long-term trends are adjusted for by design.
Detection bias for exposure	Probably Low	- Daily maximum temperature at ZIP-code-level measured using Parameter-elevation Relationships on Independent Slopes Model (PRISM) grid (4-Km) weighted by inverse of square distance between the grid and population centroid.<br>- Data were sourced from the Gridded Surface Meteorological (gridMET) dataset.
Detection bias for outcome	Probably Low	- Emergency department visits and any unscheduled hospital admissions for mental health disorders (ICD-9: 290-319; ICD-10: F00-F99)<br>- Emergency department visits and any unscheduled hospital admissions for suicidality and depression (ICD-9: 296.2-296.3, 311, V62.84; ICD-10: R45.8, F32-F33, X60-X84)<br>- Data were sourced from the California Department of Health Care Access and Information
Selective reporting bias	Probably Low	- No selective reporting bias is suspected.
Other bias	Probably Low	- No other bias is suspected.

Risk of bias summary for Ngu *et al.*, 2021.

**Risk of bias domain**	**Author's judgement**	**Support for judgement**
Confounding bias	Probably High	- NA
Detection bias for exposure	Probably Low	- Temperature data measured at the county-level derived from the ERA5 temperature data provided by European Centre for Medium-Range Weather Forecasts.
Detection bias for outcome	Probably Low	- Suicide is defined as fatal intentional self-harm derived from the WHO's mortality database.
Selective reporting bias	Probably Low	- No selective reporting bias is suspected.
Other bias	Probably Low	- No other bias is suspected.

Risk of bias summary for Nitschke *et al.,* 2007.

**Risk of bias domain**	**Author's judgement**	**Support for judgement**
Confounding bias	Probably High	- Time trend implicitly adjusted for by design.<br>- Seasonality was controlled for by excluding autumn and winter.
Detection bias for exposure	Probably High	- Daily maximum temperature ≥35°C for ≥3 consecutive days (heatwave episodes) during spring and summer.<br>- Data were sourced from the Bureau of Meteorology Kent Town.
Detection bias for outcome	Probably High	- Hospital admission for mental diseases (ICD9: 290-294-9, 580-5999; ICD-10: N00-N39)<br>- Data were sourced from the South Australian Ambulance Service.<br>- *ICD-10: N00-N39 are irrelevant to mental health.*
Selective reporting bias	Probably Low	- No selective reporting bias is suspected.
Other bias	Probably Low	- No other bias is suspected.

Risk of bias summary for Niu *et al.,* 2020.

**Risk of bias domain**	**Author's judgement**	**Support for judgement**
Confounding bias	Probably High	- Covariates included sunshine duration, precipitation, $PM_{2.5}$, SO_2, and O_3.
Detection bias for exposure	Probably High	- Daily mean temperature derived from three meteorological monitoring stations.<br>- Temperature was measured at the city level on the same day of event (lag 0), and further calculated based on both multi-single lag-day structure (lag 1, lag 2…lag 7) and period-based structure (lag01, lag02…lag07).<br>- Data were sourced from the China Meteorological Data Service Center.
Detection bias for outcome	Probably Low	- Daily number of emergency admissions associated with mental and behavioural disroders (primary cause of visit) defined based on ICD-10 codes (F00-F99).<br>- Data were sourced from the Beijing Municipal Health Commission Information Center.
Selective reporting bias	Probably Low	- No selective reporting bias is suspected.
Other bias	Probably Low	- No other bias is suspected.

Risk of bias summary for Niu *et al.,* 2023.

**Risk of bias domain**	**Author's judgement**	**Support for judgement**
Confounding bias	Probably Low	- The covariate included relative humidity (for sensitivity test).<br>- Case-crossover study: individual confounders and long-term trends are adjusted for by design.
Detection bias for exposure	Probably High	- Daily minimum temperature derived from four meteorological monitoring stations.<br>- Temperature was measured at the city-level during warm season (June-August) based on a period-based system (lag05).<br>- Data were sourced from NOAA NCDC.
Detection bias for outcome	Probably Low	- Daily emergency department visits and hospitalizations (primary diagnosis) for: mental-health disorders defined based on ICD-9 codes (290-299); anxiety defined based on ICD-9 codes (300-300.3, 300.5-300.9, 309.21, 309.81), depression defined based on ICD-9 codes (296.2-296.39, 311, 300.4, 296.9-296.99), and suicide and self-inflicted injury defined based on ICD-9 codes (E95).<br>- Data were sourced from the New York Statewide Planning and Research Cooperative System.
Selective reporting bias	Probably Low	- No selective reporting bias is suspected.
Other bias	Probably Low	- No other bias is suspected.

Risk of bias summary for Parks *et al.*, 2020.

**Risk of bias domain**	**Author's judgement**	**Support for judgement**
Confounding bias	Probably High	- NA
Detection bias for exposure	Probably Low	- Monthly temperature using gridded data from global in situ and satellite images (measured 4 times daily with a resolution of 30km).<br>- Monthly data were derived at the state-level with population as a weight factor.<br>- Data were derived from the fifth generation ECMWF atmospheric reanalysis of the global climate (ERA5).
Detection bias for outcome	Probably Low	- Suicide deaths defined based on ICD-9 (1980-1998) (E950-E959) and ICD-10 (1999-) codes (X60-X84).<br>- Data derived from the National Center for Health Statistics.
Selective reporting bias	Probably Low	- No selective reporting bias is suspected.
Other bias	Probably Low	- No other bias is suspected.

Risk of bias summary for Rahman *et al.*, 2023.

Risk of bias domain	Author's judgement	Support for judgement
Confounding bias	Probably Low	- Same lag day relative humidity<br>- Case-crossover study: individual confounders and long-term trends are adjusted for by design.
Detection bias for exposure	Probably Low	- Daily minimum and maximum temperature measured using a gridded dataset (4-Km resolution).<br>- Temperature was measured at the level of census tracts on the same day of event onset (lag 0) and further calculated based on multi-single lag-day structure (lag 1, lag 2…lag 7) using nearest grid points.<br>- Data were sourced from gridMET.
Detection bias for outcome	Probably Low	- Suicide mortality defined based on ICD-10 codes (X60-X84, Y87.0).<br>- Data were sourced from the California Department of Public Health.
Selective reporting bias	Probably Low	- No selective reporting bias is suspected.
Other bias	Probably Low	- No other bias is suspected.

Risk of bias summary for Runkle *et al.,* 2025.

**Risk of bias domain**	**Author's judgement**	**Support for judgement**
Confounding bias	Probably Low	- Covariates included population size.<br>- Retrospective matched-case study: confounders at county-level and seasonal factors are accounted for
Detection bias for exposure	Probably Low	- Daily mean temperature at county-level during summertime (May-Sep) measured using the nClimGrid data at a resolution of 1/24 of a degree (0.0417°).<br>- Temperature was measured on the same day of event (lag 0), and further calculated based on both multi-single lag-day structure (lag 1, lag 2…lag 7) and period-based structure (lag03, lag05, lag07).<br>- Data were sourced from the NOAA's nClimGrid product.
Detection bias for outcome	Probably Low	- Daily emergency department visits for major depressive disorder (ICD-9: 296.3; ICD-10: F33)<br>- Daily emergency department visits for suicidal behavior (ICD-9: E95, V628.4; ICD-10: X60-X84, R45.851, T14.91)<br>- Data were sourced from the University of North Carolina Cecil G. Sheps Center for Health Services Research.
Selective reporting bias	Probably Low	- No selective reporting bias is suspected.
Other bias	Probably Low	- No other bias is suspected.

Risk of bias summary for Stowell *et al.,* 2023.

**Risk of bias domain**	**Author's judgement**	**Support for judgement**
Confounding bias	Probably Low	- Quadratic B-spline with one internal knot placed at 50th percentile (exposure-response functions)<br>- Natural cubic B-spline with two knots placed at equal intervals on the log scale of lags up to 5 days (lag response function)<br>- Natural spline function with 3df for daily mean relative humidity and federal holidays<br>- Case-crossover study: individual confounders and long-term trends are adjusted for by design.
Detection bias for exposure	Probably Low	- Daily maximum temperature at county-level measured using Parameter-elevation Relationships on Independent Slopes Model (PRISM) grid data (4-km) weighted by population during summertime (May-Sep).<br>- Data were sourced from the National weather service.
Detection bias for outcome	Probably Low	- Emergency department visits for mental, behavioural disroders (ICD-10: F00-F99)<br>- Emergency department visits for suicidality and depression (ICD-10: R45.85-R45.87, R45.1, R45.4-R45.6, F32, F33).<br>- Data were sourced from the Optum Labs Data Warehouse.
Selective reporting bias	Probably Low	- No selective reporting bias is suspected.
Other bias	Probably Low	- No other bias is suspected.

Risk of bias summary for Trang *et al.,* 2016.

**Risk of bias domain**	**Author's judgement**	**Support for judgement**
Confounding bias	Probably Low	- Covariates included day of the week, season, and time trend
Detection bias for exposure	Probably Low	- Yearly average of maximum temperature at postcode level measured using gridded data (1km).<br>- Data were sourced from the Hanoi Mental Hospital.
Detection bias for outcome	Probably Low	- Hospital admission for mental health disorder (ICD-10: F00-F99, except F60-F69)<br>- Average maximum temperature derived from several meteorological monitoring stations
Selective reporting bias	Probably Low	- No selective reporting bias is suspected.
Other bias	Probably Low	- No other bias is suspected.

Risk of bias summary for Villeneuve *et al.,* 2023.

**Risk of bias domain**	**Author's judgement**	**Support for judgement**
Confounding bias	Probably Low	- Covariates included holidays (main analysis) and relative humidity and precipitation and air pollutants (sensitivity test)<br>- Case-crossover study: individual confounders and long-term trends are adjusted for by design.
Detection bias for exposure	Probably Low	- Daily mean temperature measured by interpolating metrics available for all postal code locations using thin-plate smoothing splines implemented in ANUSPLIN climate modeling software.<br>- Temperature was measured on the same day of event onset (lag0), and further calculated based on multi-single lag-day structure (lag 1, lag 2) and period-based system (lag01, lag02).<br>- Weights for the conditional model derived from the number of suicides deaths per day were used.<br>- Data were sourced from Canadian Urban Environmental Health Research Consortium (CANUE).
Detection bias for outcome	Probably Low	- Suicide deaths defined based on ICD-10 codes (X60-X84).<br>- Data sourced from the Canadian Vital Statistics Death Database.
Selective reporting bias	Probably Low	- No selective reporting bias is suspected.
Other bias	Probably Low	- No other bias is suspected.

Risk of bias summary for Wang *et al.,* 2018.

**Risk of bias domain**	**Author's judgement**	**Support for judgement**
Confounding bias	Probably Low	- Covariates included rainfall, day of week and holiday
Detection bias for exposure	Probably High	- Daily mean temperature measured using data from meteorological station during summertime time (May-Oct).<br>- Temperature was measured on the same day of event onset (lag0), and further calculated based on multi-single lag-day structure (lag1…lag6) and period-based system (lag01…lag06).<br>- Data were sourced from the Hefei Bureau of Meteorology
Detection bias for outcome	Probably Low	- Daily hospital admission of schizophrenia (ICD-10: F20-F29)<br>- Data were sourced from the Anhui Mental Health Centre
Selective reporting bias	Probably Low	- No selective reporting bias is suspected.
Other bias	Probably Low	- No other bias is suspected.

Risk of bias summary for Xu *et al.,* 2018.

**Risk of bias domain**	**Author's judgement**	**Support for judgement**
Confounding bias	Probably Low	- Covariates included regular participation in organised sport and other physical activities in the past 12 months, gender, age, household size, family income, religion and ethnicity.
Detection bias for exposure	Probably Low	- Yearly average of maximum temperature at postcode level measured using gridded data (1km).<br>- Data were sourced from the Australian Bureau of Meteorology.
Detection bias for outcome	Probably Low	- Mental health based on the Strengths and Difficulties Questionnaire (SDQ).<br>- Longitudinal Study of Australian Children
Selective reporting bias	Probably Low	- No selective reporting bias is suspected.
Other bias	Probably Low	- No other bias is suspected.

Risk of bias summary for Zhang *et al.,* 2020.

**Risk of bias domain**	**Author's judgement**	**Support for judgement**
Confounding bias	Probably Low	- Covariates included air pollutants, relative humidity, time, day of week, and public holiday.<br>- Case-crossover study: individual confounders and long-term trends are adjusted for by design.
Detection bias for exposure	Probably High	- Temperature was measured at the city-level on the same day of event onset (lag 0) and further calculated based on a period-based structure (lag01, lag03, lag05, lag07 days, lag09).<br>- Data were sourced from the Meteorological Data Sharing Center under the National Weather Service.
Detection bias for outcome	Probably Low	- Daily hospital outpatient visits for mental disorders defined based on ICD-10 codes [depressive disorders: F32-F33; organic mental disorders: F00-F09; anxiety: F40-F41].<br>- Data were sourced from the Psychiatric specialist hospitals.
Selective reporting bias	Probably Low	- No selective reporting bias is suspected.
Other bias	Probably Low	- No other bias is suspected.

Risk of bias summary for Zhong *et al.,* 2025.

**Risk of bias domain**	**Author's judgement**	**Support for judgement**
Confounding bias	Probably Low	- Covariates included relative humidity, wind speed, precipitation, $PM_{2.5}$, holiday, COVID status.<br>- Case-crossover study: individual confounders and long-term trends are adjusted for by design.
Detection bias for exposure	Probably Low	- Daily mean temperature using the CN05.1 grid observation dataset at a spatial resolution of 0.25° × 0.25°.<br>- Temperature was measured over lag021.<br>- Data were sourced from the CN05.1 grid observation dataset.
Detection bias for outcome	Probably Low	- Outpatient visits for mental and behavioral disorders (ICD-10: F00-F99)<br>- Outpatient visits for schizophrenia (ICD-10: F20-F21)<br>- Outpatient visits for depression (ICD-10: F32-F33)<br>- Outpatient visits for anxiety (ICD-10: F40-F41)<br>- Data were sourced from the Anhui Mental Health Center.
Selective reporting bias	Probably Low	- No selective reporting bias is suspected.
Other bias	Probably Low	- No other bias is suspected.

Risk of bias summary for Zhou *et al.,* 2023.

**Risk of bias domain**	**Author's judgement**	**Support for judgement**
Confounding bias	Probably Low	- Covariates included day of week, calendar time, holiday, wind velocity and rainfall, O_3 and $PM_{2.5}$.
Detection bias for exposure	Probably High	- Temperature was measured from a meteorological monitoring station.<br>- Temperature was measured at the city-level on the same day of event onset (lag 0), and further calculated based on a multi-single lag-day structure (lag 1, lag 2…lag14) and period-based structure (lag01, lag02…lag014).<br>- Data were sourced from the Chongqing Meteorological Bureau.
Detection bias for outcome	Probably Low	- Daily outpatient visits for depression defined based on ICD-10 codes (F32).<br>- Data were sourced from the Xinqiao Hospital and Southwest Hospital.
Selective reporting bias	Probably Low	- No selective reporting bias is suspected.
Other bias	Probably Low	- No other bias is suspected.

Risk of bias summary for Zhou *et al.,* 2024.

**Risk of bias domain**	**Author's judgement**	**Support for judgement**
Confounding bias	Probably Low	- Covariates included relative humidity, precipitation, day of week, and holiday.
Detection bias for exposure	Probably High	- Daily mean temperature sourced from the China Meteorological Data Service Centre.<br>- Temperature was measured at the city-level on the same day of event onset (lag 0), and further calculated based on a multi-single lag-day structure (lag 1, lag 2…lag14) and period-based structure (lag01, lag02…lag014).<br>- Data were sourced from the China Meteorological Data Service Centre.
Detection bias for outcome	Probably Low	- Hospital admission for schizophrenia (ICD-10: F20-F20.9).<br>- Data were sourced from the Guangxi Zhuang Autonomous Region Brain Hospital.
Selective reporting bias	Probably Low	- No selective reporting bias is suspected.
Other bias	Probably Low	- No other bias is suspected.

Supplementary File 12. Assessments of confidence in the body of evidence as per the Grading of Recommendations Assessment, Development and Evaluation (GRADE) guideline for each outcome by study design.

Initial grade[a]	Downgrading domain[bc]					Upgrading domain			Final grade of confidence in the body of evidence
	Risk of bias	Indirectness	Inconsistency	Imprecision	Publication bias	Effect magnitude	Dose-response	Confounding	
Mental disorders									
Moderate	-1	0	0	0	0	0	0	0	Low
	Serious	Not serious	Not serious	Not serious	Not serious	Not large	No	No	
Schizophrenia/other psychotic disorders									
Moderate	0	0	0	0	0	0	0	0	Moderate
	Not likely	Not serious	Not serious	Not serious	Not serious	Not large	No	No	
Depression									
Moderate	0	0	-1	0	0	0	0	0	Low
	Not likely	Not serious	Serious	Not serious	Not serious	Not large	No	No	
Anxiety									
Moderate	0	0	-1	-1	0	0	0	0	Low
	Not likely	Not serious	Serious	Serious	Not serious	Not large	No	No	
Suicide death									
Moderate	0	0	-1	0	0	0	+1	0	Moderate
	Not likely	Not serious	Serious	Not serious	Not serious	Not large	Yes	No	

[a]The initial grade is judged by four features of study design. The four features include controlled exposure, exposure prior to outcome, individual outcome data, and comparison group used. *Controlled exposure* is achieved if exposure to the environmental substance is controlled, which is generally observed in randomized controlled trial. *Exposure prior to outcome* considers if the exposure exists before an individual develops the outcome of interest. *Individual outcome data* ensures that the outcome is measured at the individual level. *Comparison group used* considers if there is a control group in the study. Studies fulfilling all the 4 features are judged to have an initial grade of high, whereas those with 3 or 2 features are judged to have a grade of moderate and low. A grade of very low is assigned to studies with 1 or fewer feature. In the present study, cohort and case-crossover studies with 3 features are judged to have an initial grade of moderate, whereas time-series and ecological studies with 2 features are judged to have an initial grade of low.

[b]The risk of bias domain is judged as *'not likely'* if most studies are evaluated to have probably low or definitely low risk of bias across all key domains (exposure, outcome, confounding). A judgement of *'very serious'* is given if most studies are judged to have probably high or definitely low risk of bias. A judgement of *'serious'* is assigned if the body of evidence do not meet either 'not likely' or 'very serious',
[c]Imprecision was evaluated based on the confidence intervals of the effect estimates. A judgement of *'not serious'* is assigned to studies with the ratio between upper and lower 95% confidence intervals for most studies is <10, whereas a judgement of *'very serious'* is assigned when the ratio is ≥10.

Supplementary File 13. Sensitivity analysis using the DerSimonian-Laird random-effects model.

Meta-analysis of associations of exposure to high temperature with hospital visits or hospitalizations for mental health sequelae among children and adolescents relative to low temperature.

Outcome	Estimate count [study count]	Relative risk (95% CI)	τ^2	I^2 (%)
**Mental health sequelae**				
Mental health disorders[a]	10 [8]	1.14 (1.06, 1.23)	0.01	86
Schizophrenia or other psychotic disorders[b]	6 [6]	1.13 (1.01, 1.27)	0.01	73
Depression[c]	5 [4]	1.18 (1.04, 1.33)	0.01	59
Anxiety[d]	4 [3]	1.15 (0.87, 1.51)	0.03	33
Composite mental illnesses (all sequelae)[e]	17 [15]	1.12 (1.06, 1.18)	0.01	82
**Suicide**	5 [5]	1.01 (1.00, 1.02)	0.00	66

[a]ICD-10: F00-F99 or ICD-9: 290.xx-319.xx.
[b]ICD-10: F20-F29 or ICD-10-CM: 293.8, 295, 297, 298.
[c]ICD-10: F32-F33 or ICD-9: 296.2-296.39, 296.9-296.99, 300.4, 311.
[d]ICD-10: F40-F41 or ICD-9: 300-300.3, 300.5-300.9, 309.21, 309.81.
[e]The definition of composite mental illnesses comprised all sequelae including mental health disorders, schizophrenia or psychotic disorders, depression and anxiety.
Abbreviations: CI, confidence intervals; CM, clinical modification; ICD, International Classification of Diseases; pc, percentile; temp., temperature

Supplementary File 14. Funnel plots examining biases.

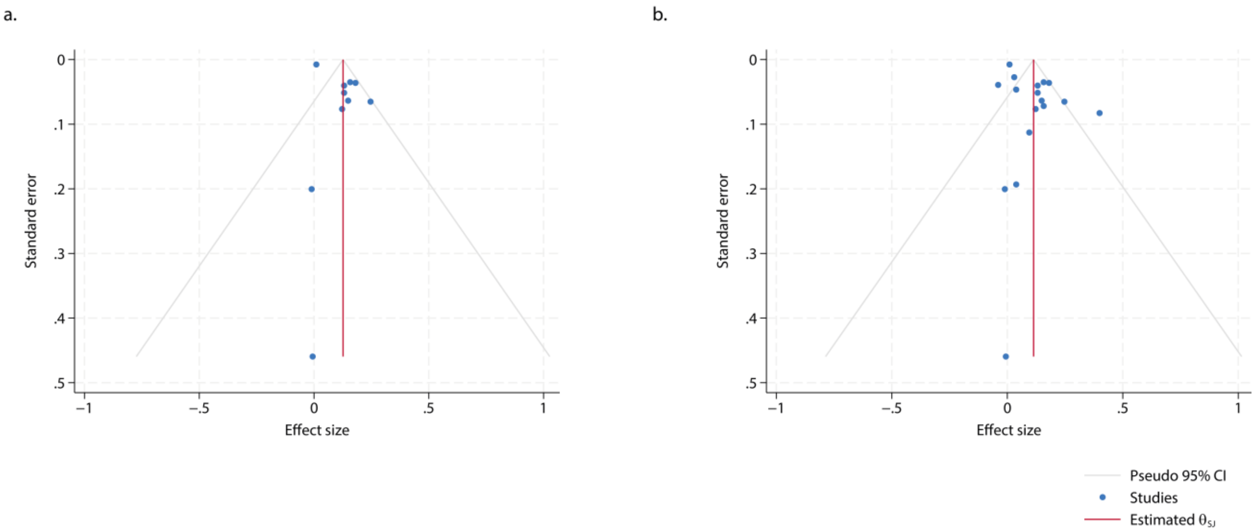

Standard error plotted against the effect size for studies examining associations of temperature with hospital visits or hospitalizations for (a) mental health disorders and (b) composite mental health illnesses among children and adolescents. The effect sizes were derived from studies on associations of exposure to greater temperature increment with suicide deaths. Each dot represents the estimate of a study.

References

1 Basu, R., Gavin, L., Pearson, D., Ebisu, K. & Malig, B. Examining the Association Between Apparent Temperature and Mental Health-Related Emergency Room Visits in California. *American Journal of Epidemiology* **187**, 726-735, doi:10.1093/aje/kwx295 (2018).

2 Bernstein Aaron, S. *et al.* Warm Season and Emergency Department Visits to U.S. Children's Hospitals. *Environmental Health Perspectives* **130**, 017001, doi:10.1289/EHP8083 (2022).

3 Chan, E. Y. Y. *et al.* Association between Ambient Temperatures and Mental Disorder Hospitalizations in a Subtropical City: A Time-Series Study of Hong Kong Special Administrative Region. *International Journal of Environmental Research and Public Health* **15** (2018).

4 da Silva, I., de Almeida, D. S., Hashimoto, E. M. & Martins, L. D. Risk assessment of temperature and air pollutants on hospitalizations for mental and behavioral disorders in Curitiba, Brazil. *Environmental Health* **19**, 79, doi:10.1186/s12940-020-00606-w (2020).

5 Isaksen, T. B. *et al.* Increased mortality associated with extreme-heat exposure in King County, Washington, 1980–2010. *International Journal of Biometeorology* **60**, 85-98, doi:10.1007/s00484-015-1007-9 (2016).

6 Niu, Y. *et al.* Short-term effect of apparent temperature on daily emergency visits for mental and behavioral disorders in Beijing, China: A time-series study. *Science of The Total Environment* **733**, 139040, doi:10.1016/j.scitotenv.2020.139040 (2020).

7 Parks, R. M. *et al.* Anomalously warm temperatures are associated with increased injury deaths. *Nature Medicine* **26**, 65-70, doi:10.1038/s41591-019-0721-y (2020).

8 Zhou, Y. *et al.* The role of extreme high humidex in depression in chongqing, China: A time series-analysis. *Environmental Research* **222**, 115400, doi:10.1016/j.envres.2023.115400 (2023).

9 Mullins, J. T. & White, C. Temperature and mental health: Evidence from the spectrum of mental health outcomes. *Journal of Health Economics* **68**, 102240, doi:10.1016/j.jhealeco.2019.102240 (2019).

10 Wang, S. *et al.* Effect of increasing temperature on daily hospital admissions for schizophrenia in Hefei, China: a time-series analysis. *Public Health* **159**, 70-77, doi:10.1016/j.puhe.2018.01.032 (2018).

11 Zhou, Q. *et al.* Immediate and delayed effects of environmental temperature on schizophrenia admissions in Liuzhou, China, 2013–2020: a time series analysis. *International Journal of Biometeorology* **68**, 843-854, doi:10.1007/s00484-024-02629-1 (2024).

12 Trang, P. M., Rocklöv, J., Giang, K. B., Kullgren, G. & Nilsson, M. Heatwaves and Hospital Admissions for Mental Disorders in Northern Vietnam. *PLOS ONE* **11**, e0155609, doi:10.1371/journal.pone.0155609 (2016).

13 Nitschke, M., Tucker, G. R. & Bi, P. Morbidity and mortality during heatwaves in metropolitan Adelaide. *Medical Journal of Australia* **187**, 662-665, doi:10.5694/j.1326-5377.2007.tb01466.x (2007).

14 Kim, Y. *et al.* Suicide and Ambient Temperature in East Asian Countries: A Time-Stratified Case-Crossover Analysis. *Environmental Health Perspectives* **124**, 75-80, doi:10.1289/ehp.1409392 (2016).

15 Niu, L. *et al.* Temperature and mental health–related emergency department and hospital encounters among children, adolescents and young adults. *Epidemiology and Psychiatric Sciences* **32**, e22, doi:10.1017/S2045796023000161 (2023).

16 Rahman, M. M. *et al.* Ambient temperature and air pollution associations with suicide and homicide mortality in California: A statewide case-crossover study. *Science of The Total Environment* **874**, 162462, doi:10.1016/j.scitotenv.2023.162462 (2023).

17 Villeneuve, P. J. *et al.* Daily changes in ambient air pollution concentrations and temperature and suicide mortality in Canada: Findings from a national time-stratified case-crossover study. *Environmental Research* **223**, 115477, doi:10.1016/j.envres.2023.115477 (2023).
18 Zhang, S. *et al.* The effect of temperature on cause-specific mental disorders in three subtropical cities: A case-crossover study in China. *Environment International* **143**, 105938, doi:10.1016/j.envint.2020.105938 (2020).
19 Cohen, G. *et al.* Daily temperature variability and mental health-related hospital visits in New York State. *Environmental Research* **257**, 119238, doi:10.1016/j.envres.2024.119238 (2024).
20 He, W.-Q. *et al.* Extreme Heat Stress and Unplanned Hospital Admissions. *Pediatrics* **155**, e2024068183, doi:10.1542/peds.2024-068183 (2024).
21 Ndovu, A. *et al.* Spatial Variation in the Association between Extreme Heat Events and Warm Season Pediatric Acute Care Utilization: A Small-Area Assessment of Multiple Health Conditions and Environmental Justice Implications in California (2005–2019). *Environmental Health Perspectives* **133**, 017010, doi:10.1289/EHP14236 (2025).
22 Zhong, Z. *et al.* The impact of different types of extreme temperature events on mental disorders: A case-crossover study in Anhui Province, China. *Environmental Research* **277**, 121526, doi:10.1016/j.envres.2025.121526 (2025).
23 Stowell, J. D. *et al.* Warm-season temperatures and emergency department visits among children with health insurance. *Environmental Research: Health* **1**, 015002, doi:10.1088/2752-5309/ac78fa (2023).
24 Komulainen, K. *et al.* Climatic exposures in childhood and the risk of schizophrenia from childhood to early adulthood. *Schizophrenia Research* **248**, 233-239, doi:10.1016/j.schres.2022.09.013 (2022).
25 Xu, Y., Wheeler, S. A. & Zuo, A. Will boys' mental health fare worse under a hotter climate in Australia? *Population and Environment* **40**, 158-181, doi:10.1007/s11111-018-0306-6 (2018).
26 Hu, J. *et al.* Associations of exposure to heatwaves with depression and anxiety among adolescents: A cross-sectional study of the Chinese adolescent health survey. *Journal of Affective Disorders* **387**, 119499, doi:10.1016/j.jad.2025.119499 (2025).
27 Runkle, J. D. *et al.* Assessing the impact of heatwaves on emergency visits for major depression and suicidal ideation in youth with attention-deficit/hyperactivity disorder. *PLOS Mental Health* **2**, e0000444, doi:10.1371/journal.pmen.0000444 (2025).
28 Florido Ngu, F., Kelman, I., Chambers, J. & Ayeb-Karlsson, S. Correlating heatwaves and relative humidity with suicide (fatal intentional self-harm). *Scientific Reports* **11**, 22175, doi:10.1038/s41598-021-01448-3 (2021).
